# Engineered Extracellular Vesicles Driven by Erythrocytes Ameliorate Bacterial Sepsis by Iron Recycling, Toxin Clearing and Inflammation Regulation

**DOI:** 10.1002/advs.202306884

**Published:** 2024-01-21

**Authors:** Yan Li, Guanlin Qu, Geng Dou, Lili Ren, Ming Dang, Huijuan Kuang, Lili Bao, Feng Ding, Guangzhou Xu, Zhiyuan Zhang, Chi Yang, Shiyu Liu

**Affiliations:** ^1^ National Center for Stomatology National Clinical Research Center for Oral Diseases Shanghai Key Laboratory of Stomatology Research Unit of Oral and Maxillofacial Regenerative Medicine Chinese Academy of Medical Sciences Department of Oral Surgery Shanghai Ninth People's Hospital Shanghai Jiao Tong University School of Medicine College of Stomatology Shanghai Jiao Tong University Shanghai 200011 China; ^2^ State Key Laboratory of Oral & Maxillofacial Reconstruction and Regeneration National Clinical Research Center for Oral Diseases Shaanxi Key Laboratory of Stomatology Department of Prosthodontics School of Stomatology The Fourth Military Medical University Shaanxi 710032 China; ^3^ State Key Laboratory of Oral & Maxillofacial Reconstruction and Regeneration National Clinical Research Center for Oral Diseases Shaanxi International Joint Research Center for Oral Diseases Center for Tissue Engineering School of Stomatology The Fourth Military Medical University Shaanxi 710032 China; ^4^ School of Dentistry University of Michigan Ann Arbor MI 48109 USA

**Keywords:** bacterial infection, bioengineering, biotechnology, drug delivery, extracellular vesicles

## Abstract

Sepsis poses a significant challenge in clinical management. Effective strategies targeting iron restriction, toxin neutralization, and inflammation regulation are crucial in combating sepsis. However, a comprehensive approach simultaneously targeting these multiple processes has not been established. Here, an engineered apoptotic extracellular vesicles (apoEVs) derived from macrophages is developed and their potential as multifunctional agents for sepsis treatment is investigated. The extensive macrophage apoptosis in a *Staphylococcus aureus*‐induced sepsis model is discovered, unexpectedly revealing a protective role for the host. Mechanistically, the protective effects are mediated by apoptotic macrophage‐released apoEVs, which bound iron‐containing proteins and neutralized α‐toxin through interaction with membrane receptors (transferrin receptor and A disintegrin and metalloprotease 10). To further enhance therapeutic efficiency, apoEVs are engineered by incorporating mesoporous silica nanoparticles preloaded with anti‐inflammatory agents (microRNA‐146a). These engineered apoEVs can capture iron and neutralize α‐toxin with their natural membrane while also regulating inflammation by releasing microRNA‐146a in phagocytes. Moreover, to exploit the microcosmic movement and rotation capabilities, erythrocytes are utilized to drive the engineered apoEVs. The erythrocytes‐driven engineered apoEVs demonstrate a high capacity for toxin and iron capture, ultimately providing protection against sepsis associated with high iron‐loaded conditions. The findings establish a multifunctional agent that combines natural and engineered antibacterial strategies.

## Graphical abstract

Here, apoptotic extracellular vesicles (apoEVs) are found to bind iron‐containing proteins and neutralize α‐toxin by membrane receptors. The apoEVs are further engineered by incorporating material loaded with anti‐inflammatory microRNA‐146a and are connected to erythrocytes to harness their movement and rotation abilities in circulation. The engineered erythrocytes‐driven apoEVs show high capacity to capture toxin and iron, regulate inflammation and protect against sepsis.

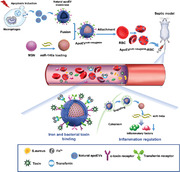

## 1 Introduction

Sepsis represents a major global health concern, causing significant morbidity and mortality worldwide, with an estimated 48.9 million new cases and 11 million deaths yearly.^[^ ^1^ ^]^ Despite these alarming statistics, the treatment options for sepsis remain limited. In bacterial infection, the availability of circulating iron in the blood is critical in the course of infection. Meanwhile, bacterial toxin contributed significantly to the associated mortality.^[^ ^2^ ^]^ Therefore, simultaneously restricting iron access for bacteria and neutralizing their toxins could potentially alleviate the impact of severe bacterial infections and benefit the patients. Although multiple strategies have been employed to restrict iron^[^ ^3^ ^]^ or neutralize toxins,^[^ ^4^ ^]^ a single‐target therapeutic strategy may not efficiently treat the disease, especially under excessive inflammation during sepsis. Therefore, alternative approaches that simultaneously target the irons, toxins and inflammation are highly desirable for effective sepsis treatment.

Host organisms employ multiple mechanisms of resistance to defend against microbial threats.^[^ ^5^ ^]^ A comprehensive understanding of these cellular responses triggering the production of defensosomes during infection may leverage their unique properties to combat bacteria and offer novel therapeutic strategies. Cell death, including pyroptosis, necrosis and apoptosis, is increasingly recognized as a crucial host defense mechanism.^[^ ^6^ ^]^ Previous studies have unveiled the utility of pyroptosis or necrosis during infection to destroy intracellular niches, and subsequently coordinate an appropriate innate immune response, which leads to bacteria elimination via pore‐induced intracellular trap or neutrophil extracellular traps.^[^ ^7^ ^]^ However, further research is needed to fully understand how apoptosis contributed to the defense against infection in vivo. Apoptosis converts cells into apoptotic extracellular vesicles (apoEVs),^[^ ^8^ ^]^ yet it remains unknown whether apoEVs released during bacterial infection could generate anti‐bacterial response. Surprisingly, in our study we found that *Staphylococcus aureus* (*S. aureus*), a predominant bacterium associated with sepsis, triggered macrophage apoptosis and led to production of macrophage‐derived apoEVs into circulation. These apoEVs confer benefits by serving as decoys to capture iron‐containing proteins and simultaneously neutralize bacterial toxins through surface receptors. These findings suggest that apoEVs released by cells under bacterial attacks could be employed as novel therapeutic tool for sepsis.

The successful administration of apoEVs in vivo to capture specific molecules require sufficient contact with these molecules. Prolonging the circulating half‐life of EVs is challenging to avoid rapid uptake by phagocytes after in vivo administration. Moreover, the high viscosity of biological fluids^[^ ^9^ ^]^ and the small size of EVs greatly limit their microcosmic movements, resulting in low efficiency of EV‐molecule interaction. Bacteria have evolved various ways to move within highly viscous biological fluids for efficient capture of nutrient molecules.^[^ ^10^ ^]^ Therefore, new strategies are needed to endow the EVs with movement capacity after being administrated in host circulation, thereby significantly enhancing their ability to capture iron‐containing proteins and neutralize toxins. Furthermore, natural apoEVs contain multiple inner components unrelated to the therapeutic goals,^[^ ^8^, ^11^ ^]^ which needs to be removed. Thus, engineering modifications are required to address these limitations associated with natural apoEVs.

Here, we developed engineered apoEVs with multiple therapeutic functions and investigated their potential anti‐infection agent for sepsis therapy. Specially, we combined the natural apoEVs membrane with mesoporous silica nanoparticles (MSNs) preloaded with anti‐inflammatory agents (microRNA‐146a) to construct engineered apoEVs. The resulting engineered apoEVs captured iron‐containing proteins and neutralized bacterial toxins with their natural membrane while also regulating inflammation through controlled release of microRNA‐146a in phagocytes that engulf apoEVs. Moreover, to enhance the microcosmic moving ability of the engineered EVs, we tethered them to erythrocytes to harness the movement and rotation abilities of erythrocytes in blood circulation. The engineered erythrocytes‐driven EVs showed a high capacity to capture toxin and iron, and efficiently regulated the inflammation, ultimately providing protection against infection and tissue damage in high iron‐loaded sepsis.

## 2 Results

### 2.1 Bacteria‐Induced Host Macrophage Apoptosis Generated Protecting Effects on Sepsis

**Figure 1:**
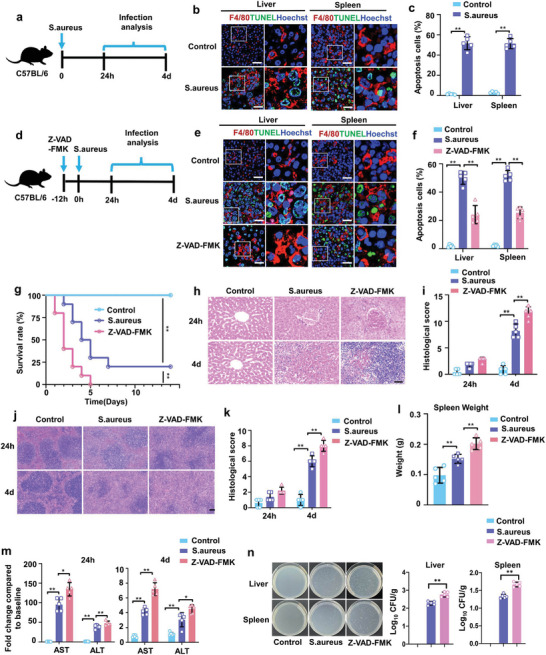
Bacteria‐induced host macrophage apoptosis generate protecting effects on sepsis. a) Schematic diagram of the experimental procedure of bacterial infection. *S. aureus* was intravenously administered to C57BL/6 mice. The control mice and *S. aureus*‐infected mice were euthanized at 24 h or 4 days after infection for further analysis. b) Immunostaining showed that *S. aureus* injection induced apoptosis of macrophages in liver or spleen of mice. Scale bar, 100 µm. c) The percentage of the apoptosis cells population. n = 5 mice. d) Schematic diagram of the experimental design to test the role for apoptosis of macrophage in sepsis. Empty liposomes or Z‐VAD‐FMK‐encapsulated liposomes were injected intravenously into mice 12 h before *S. aureus* injection. After 24 h or 4 days, the mice were euthanized for further analysis. e) Immunostaining showed that Z‐VAD‐FMK treatment prevent macrophages apoptosis in liver or spleen of C57BL/6 mice. Scale bar, 100 µm. f) The percentage of the apoptosis cells population. n = 5 mice. g) Survival rate of uninfected mice, infected mice, or infected mice receiving Z‐VAD‐FMK pretreatment. n = 10 mice. h,i) H&E staining of representative liver sections h) and the associated histological scores i) at 24 h or 4 days after infection. Scale bar, 50 µm. n = 5 mice. j,k) H&E staining of representative spleen sections j) and the associated histological scores k) at 24 h or 4 days after infection. Scale bar, 50 µm. n = 5 mice. l) The spleen weight at 4 days after infection. n = 5 mice. m) ALT and AST levels in the serum of uninfected mice, infected mice, or infected mice receiving apoptotic Z‐VAD‐FMK. n = 5 mice. All results are representative of the data generated in at least three independent experiments. n) Viable count of *S. aureus* in liver or spleen tissue at 12 h after infection. n = 5 mice. For c,f,i,k–n) data are represented as the mean ± s.d. For c) statistical significance was assessed by unpaired two‐tailed Student's t test. For f,i,k–n) statistical significance was assessed by one‐way ANOVA with Tukey's post hoc test. For (g) statistical significance was assessed by the log‐rank test. ^*^ *p* < 0.05, ^**^ *p* < 0.01.

Pathogen‐triggered cell death has important consequences on sepsis progression.^[^ ^7^, ^12^ ^]^ We first determined the occurrence of apoptosis of host cells in response to *Staphylococcus aureus* (*S. aureus*) infection. Since bacterial components trigger apoptosis in macrophages during an innate defense process,^[^ ^13^ ^]^ we evaluated apoptosis of macrophages in a *S. aureus* infection model (**Figure** **1**a). The TUNEL staining showed that *S. aureus* infection induced substantial host macrophage apoptosis in lung or spleen of mice (Figure 1b,c), suggesting macrophage apoptosis might play important roles in S. aureus‐induced pathogenesis. Meanwhile, we accessed the shortened survival (Figure S1a, Supporting Information) and examined the histological changes in *S*. aureus‐infected mice. Histologic examination of the liver or spleen showed severe tissue injury at 4 days after *S. aureus* infection, which was shown by elevated histological scores (Figure S1b,c, Supporting Information). In parallel, we measured aminotransferase (AST) and alanine aminotransferase (ALT) levels in serum (Figure S1d, Supporting Information), which were significantly elevated in *S. aureus*‐infected mice. Splenomegaly is common following bacterial infection. Accordingly, we found *S. aureus* infection increased the spleen weight (Figure S1e, Supporting Information).

We then determined the role of apoptosis of macrophage in sepsis. We used caspase activity inhibitor Z‐VAD‐FMK delivered by liposome to inhibit apoptosis of macrophages in the presence of *S. aureus* infection (Figure 1d). As expected, Z‐VAD‐FMK treatment inhibited macrophages apoptosis indicated by TUNEL staining (Figure 1e,f). While it is surprising that the administration of anti‐apoptotic drug Z‐VAD‐FMK in host macrophages resulted in worsened mortality (Figure 1g). Compared with untreated septic mice, Z‐VAD‐FMK‐pretreated septic mice showed lower survival rate and died within 5 days. Regarding the histological analysis, at 4 days post‐infection, the septic mice pretreated with Z‐VAD‐FMK developed more substantial liver (Figure 1h) and spleen injury (Figure 1j) along with significantly increased liver (Figure 1i) and spleen injury scores (Figure 1k) and more enlarged spleen (Figure 1l), compared with untreated septic mice. Consistently, pretreatment with Z‐VAD‐FMK significantly enhanced serum ALT and AST levels in the infected mice (Figure 1m) and increased the bacteria number in target organs (liver and spleen) (Figure 1n). These data demonstrated that the apoptosis of macrophage plays an unexpected protective role during infection.

### 2.2 ApoEVs Released by Apoptotic Macrophage Generated Protecting Effects on Sepsis

**Figure 2:**
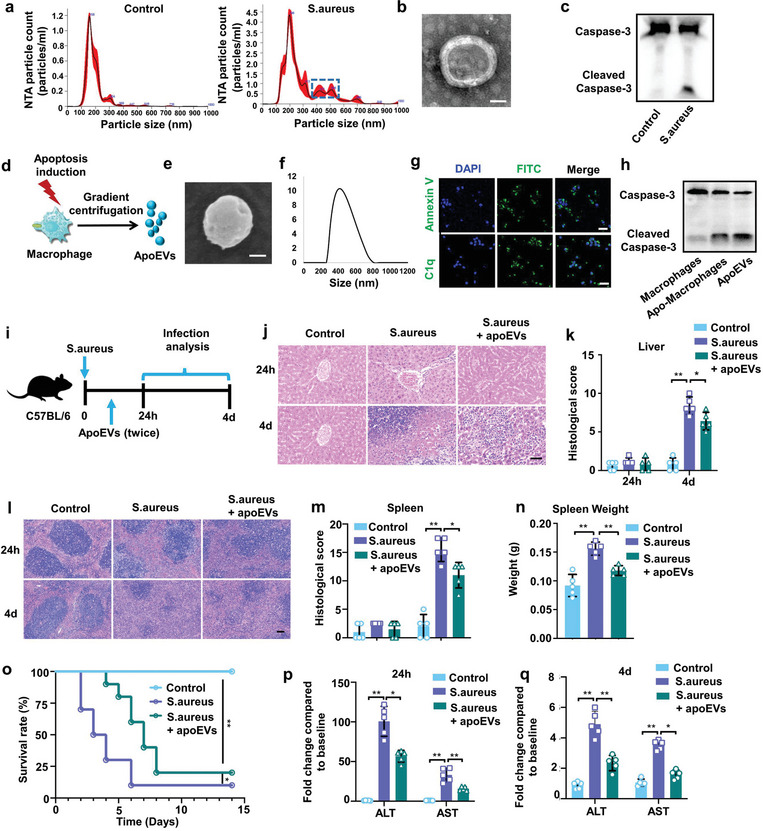
ApoEVs released by apoptotic macrophages generate protecting effects on sepsis. a) Nanoparticle tracking analysis (NTA) showing the variety of extracellular vesicles present in serum of mice. Representative NTA profiles from uninfected and infected mice are shown. b) Representativae TEM image of apoEVs isolated from serum of infected mice. Scale bar, 200 nm. c) Representative western blot analyzing caspase‐3 levels in EVs isolated from uninfected or infected mice. d) Schematic diagram of the experimental procedure to induce macrophages apoptosis. Macrophages were incubated with STS for 4 h. The apoEVs were isolated by a gradient centrifugation protocol. e) Representative SEM image of apoEVs isolated from apoptotic macrophages. Scale bar, 200 nm. f) Size distribution of apoEVs. g) Annexin V and C1q staining of apoEVs. Scale bars, 20 µm. h) The expression of caspase‐3 and cleaved caspase‐3 in macrophages, apoptotic macrophages or apoEVs assessed by western blot. i) Schematic diagram of the in vivo experimental procedure. *S. aureus* infected mice were injected with apoEVs at 1 and 6 h after infection. j,k) H&E staining of representative liver sections j) and the associated histological scores k) at 24 h or 4 days after apoEVs injection. Scale bar, 50 µm. n = 5 mice. l,m) H&E staining of representative spleen sections l) and the associated histological scores m) at 24 h or 4 days after apoEVs injection. Scale bar, 50 µm. n = 5 mice. n) The spleen weight at 4 days after apoEVs injection. n = 5 mice. o) Survival of uninfected mice, infected mice, or infected mice receiving apoEVs. n = 10 mice. p,q) ALT and AST levels in the serum of uninfected mice, infected mice, or infected mice receiving apoEVs. n = 5 mice. All results are representative of the data generated in at least three independent experiments. For (k,m,n,p,q) data are represented as the mean ± s.d. For (k,n,p,q) statistical significance was assessed by one‐way ANOVA with Tukey's post‐hoc test. For (m) statistical significance was assessed by Kruskal–Wallis test. For (n) statistical significance was assessed by the log‐rank test. ^*^ *p* < 0.05, ^**^ *p* < 0.01.

Apoptosis is widely known as programmed cell death that led to the formation of apoEVs. To characterize the population of extracellular vesicles in circulation after infection, nanoparticle tracking analysis (NTA) was performed in serum samples collected from *S. aureus*‐infected or uninfected mice. NTA revealed that the size of circulating EVs in serum of *S. aureus*‐infected mice was significantly larger than that in uninfected control mice (**Figure** **2**a). Specifically, the mean diameter of serum EVs from control mice was 175.4 +/− 6.8 nm, while *S. aureus* infection increased the mean diameter to 293.3 +/− 2.5 nm. Importantly, NTA analysis demonstrated distinct peaks at 400–600 nm exclusively in *S. aureus*‐infected mice serum but not in controls. To further characterize isolated EVs from serum of infected mice, a combination of transmission electron microscopy (TEM) and western blot were applied. TEM confirmed the presence of structures typical of EVs in the size range of 400–600 nm (Figure 2b). Western blot demonstrated that the EVs from *S. aureus*‐infected mice, but not the control EVs, were positive for apoptosis‐specific marker cleaved caspase‐3 (Figure 2c). These data verified that *S. aureus* infection led to the production of apoEVs in circulation.

After consideration of the roles of apoEVs responding to infections, we examined the evidence for the contribution of macrophage‐derived apoEVs in *S. aureus* infection. We produced apoEVs from apoptotic bone marrow‐derived macrophages (BMDMs) induced by in vitro treatment of staurosporine (STS), a broad‐spectrum protein kinase inhibitor that has been used extensively to induce apoptosis^[^ ^14^ ^]^ (Figure 2d). Scanning electron microscopy (SEM) showed that the size of the apoptotic macrophages‐produced EVs was ≈500 nm (Figure 2e), which was consistent with the dynamic light scattering (DLS) data (Figure 2f). Immunostaining demonstrated that the isolated EVs expressed apoEV‐associated surface markers Annexin V and C1q (Figure 2g). Consistently, western blot showed that the isolated EVs displayed high expression level of cleaved caspase‐3, indicating the successful apoptosis induction (Figure 2h). These data suggested that the strategy employed for preparing macrophage‐derived apoEVs is feasible and valid.

It has been suggested that host uses programmed immune cell death to fight infections.^[^ ^7^, ^15^ ^]^ Given that we found the production of apoEVs is a feature of host response to infection, we evaluated the therapeutic potential of apoEVs and tested their effects on *S. aureus*‐induced sepsis model. ApoEVs derived from in vitro cultured apoptotic BMDMs were intravenously administered after disease onset (Figure 2i). We found that apoEVs improved survival and ameliorated tissue injury under sepsis. Specially, apoEVs effectively improved hepatopathological changes including attenuated liver injury area and inflammatory infiltration (Figure 2j), alleviated pathological lesion and improved splenic structure in the spleen (Figure 2l). Meanwhile, treatment with apoEVs significantly reduced liver (Figure 2k) and spleen injury scores (Figure 2m) and alleviated spleen enlargement (Figure 2n). Importantly, apoEVs treatment also improved survival under sepsis (Figure 2o). While most of the septic mice died within 6 days, apoEVs‐treated septic mice had higher survival rate. Consistent with amelioration of liver injury, apoEVs treatment reduced liver AST and ALT levels (Figure 2p,q). These data demonstrated that intravenous injection of apoEVs protects the host against infection.

### 2.3 ApoEVs Released by Apoptotic Macrophages Captured Iron and Bacterial Toxin

**Figure 3:**
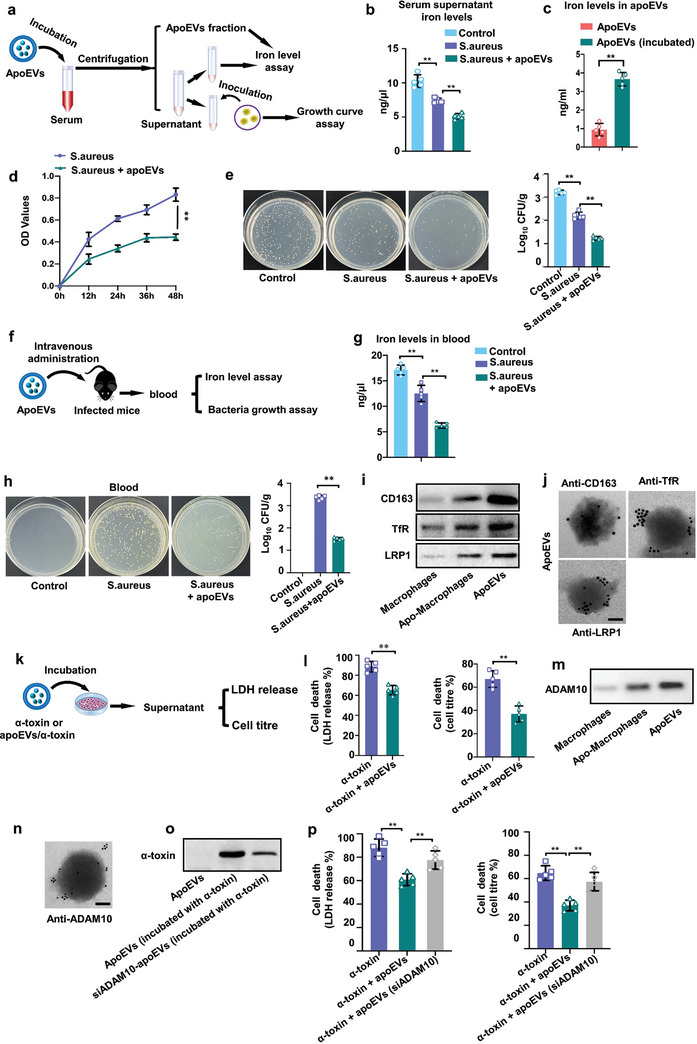
ApoEVs released by apoptotic macrophages bind iron and protected against bacterial toxin. a) Schematic diagram of the experimental design to test the ability of apoEVs to bind iron in vitro. For in vitro studies, infected mice serum was incubated with apoEVs. Then the apoEVs were isolated by gradient centrifugation protocol. The serum supernatant was used for further analysis. b) Total iron levels in the serum supernatant. n = 5. c) Iron levels in apoEVs fractions after incubation with EVs‐depleted infected serum. n = 5. d) Growth curve of *S. aureus* in serum supernatant. n = 5. e) Viable count of *S. aureus* in serum supernatant. n = 5. f) Schematic diagram of the in vivo experimental procedure. For in vivo studies, infected mice received apoEVs (50 µg of membrane protein). The blood was used for further analysis. g) Total iron levels in blood at 12 h after *S. aureus* injection. h) Viable count of *S. aureus* in blood at 12 h after *S. aureus* injection. n = 5 mice. i) Western blot analysis of CD163, TfR and LRP1 expressions in macrophages, apoptotic macrophages, and apoEVs. j) Immunoelectron microscopy detection of CD163, TfR, LRP1 in apoEVs. Scale bar, 50 nm. k) Schematic diagram of the experimental design to test cell death. α‐toxin or apoEVs/α‐toxin mixture was incubated with A549 target cells. Cell viability was then determined either by LDH release or by metabolic activity. l) Cell death following treatment with α‐toxin together with apoEVs. n = 5. m) Western blot analysis of ADAM10 expression in macrophages, apoptotic macrophages, or in apoEVs. n) Immunoelectron microscopy detection of ADAM10 antibodies in apoEVs. Scale bar, 50 nm. o) Western blot analysis of α‐toxin neutralizing efficacy in apoEVs isolated from WT or ADAM10 knockdown cells. p) Cell death following treatment with *S. aureus* together with apoEVs (siADAM10) (apoptotic EVs derived from ADAM10 knockdown cells). n = 5. All results are representative of the data generated in at least three independent experiments. For (b,e,g,h,p) data are presented as means ± s.d., statistical significance was assessed by one‐way ANOVA with Tukey's post‐hoc test. For (c,l) statistical significance was assessed by unpaired two‐tailed Student's t test. ^*^ *p* < 0.05, ^**^ *p* < 0.01.

We next determined how apoEVs defend against *S. aureus* infection. Immediate restriction of iron has been viewed as a critical innate defense mechanism against bacterial infection.^[^ ^16^ ^]^ We wondered whether apoEVs directly bound iron and expressed these iron‐related receptors. To determine whether these apoEVs bound iron to sequester iron in serum for inhibiting bacterial growth. ApoEVs were added to EVs‐depleted serum from *S. aureus*‐infected mice. After incubation, the added apoEVs were isolated by centrifugation. Then, both isolated apoEVs and serum supernatant were collected for iron amount analysis (**Figure** **3**a). The results showed that the addition of apoEVs decreased iron levels in serum (Figure 3b). Furthermore, the apoEVs used for incubation had higher iron amount (Figure 3c). There data indicate that apoEVs had iron capture abilities.

The *S. aureus* is a highly iron‐dependent bacterial pathogen.^[^ ^17^ ^]^ As apoEVs induced significant decrease in iron in serum supernatant, we then determined whether this serum supernatant was unfavorable for bacterial growth after apoEVs binding iron. Serum supernatant after incubation with apoEVs was inoculated with *S. aureus* and significant differences in their growth curves were observed, including in the overall pattern of the growth curve, lag phase and peak doubling time (Figure 3d). We found that, *S. aureus* grew more slowly in the serum supernatant which was incubated with apoEVs (Figure 3e). We then determined whether the iron‐binding capability of apoEVs could inhibit bacterial growth in sepsis by enhancing host iron recycling. The mice were injected intravenously with apoEVs or an equal volume of PBS after *S. aureus* infection (Figure 3f). We found that administration of apoEVs led to a marked drop in serum iron of infected mice (Figure 3g) and decreased number of *S. aureus* in blood (Figure 3h).

During bacterial infection, to restrict the bacteria from iron acquisition, macrophages take up iron via receptors including hemoglobin‐haptoglobin receptor (CD163), transferrin receptor (TfR) and lipoprotein receptor‐related protein 1 (LRP1). Given that we found apoEVs participating in iron sequestration, we wondered whether apoEVs expressed these iron‐related receptors. We found that after apoptosis induction, the expressions of CD163, TfR and LRP1 were upregulated in BMDMs, and showed enrichment in apoEVs (Figure 3i). Further determination of CD163, TfR and LRP1 localization was performed by immunoelectron microscopy, and the results showed that CD163, TfR and LRP1 were present on the surface of apoEVs (Figure 3j). To verify the role of TfR in this mechanism, we inhibited TfR expression in apoptotic BMDMs with TfR siRNA. We found that the expression of TfR was evidently decreased in apoEVs after TfR siRNA knockdown (Figure S2a, Supporting Information). Moreover, inhibition of TfR expression was accompanied by weakened ability of binding irons (Figure S2b, Supporting Information) and inhibiting *S. aureus* growth (Figure S2c, Supporting Information), supporting the mechanism by which apoEVs could capture irons via membrane receptors.

Although we found that administration of apoEVs could restrict iron accessibility and consequently protect host against infection, the protective effects generated by apoEVs during infection require further clarification. The production of pore‐forming α‐toxin that disrupts the plasma membrane of host cells is a common virulence strategy for bacterial pathogens such as *S. aureus*. Next, we tested whether apoEVs could serve as a host response to bind α‐toxin (Figure 3k). We found that adding apoEVs improved the viability of the cells exposed to α‐toxin (Figure 3l), indicating that apoEVs were able to protect cells from *S. aureus* pore‐forming α‐toxin toxicity. A disintegrin and metalloprotease 10 (ADAM10) has been identified as α‐toxin receptor. Surface level of ADAM10 critically contributes to α‐toxin‐dependent pathology of *S. aureus* infections in mice.^[^ ^18^ ^]^ We found that the expressions of ADAM10 were significantly upregulated and enriched in apoEVs (Figure 3m). Immunoelectron microscopy showed that ADAM10 was present on the surface of apoEVs (Figure 3n). Next, we tested whether these apoEVs could serve as a host response to bind α‐toxin. Western blot revealed that apoEVs protected cells by binding α‐toxin after the apoEVs/α‐toxin mixture incubation for 1 h. Inhibition of ADAM10 expression on apoEVs by siRNA (Figure S2d, Supporting Information) in parent BMDMs impaired apoEVs from binding α‐toxin (Figure 3o). Moreover, inhibition of ADAM10 by siRNA was accompanied by weakened ability to protect human lung epithelial cells (A549) target cells from toxicity (Figure 3p), supporting the mechanism by which apoEVs could bind α‐toxin via membrane receptors. Here, these data indicated that apoEVs protect host cells by serving as scavengers that bind α‐toxin.

### 2.4 Construction and Characterization of Engineered ApoEVs

Great interest in the therapeutic potential of apoEVs was generated when it was demonstrated that apoEVs ameliorated tissue injury and improved survival during *S. aureus* infection. Despite the remarkable utility of native apoEVs, the heterogeneity and complicated components of apoEVs reduce therapeutic efficacy and bring safety concerns. Further bioengineering refinement is required to address clinical and commercial limitations. Since the presence of specific receptors on the surfaces of apoEVs has been found to allow for binding irons or toxins, here, apoEV membrane‐coated nanoparticles (apoEV^@MSN^) were developed by fusing natural apoEVs cell membranes onto a synthetic core (mesoporous silica nanoparticle, MSN). Mesoporous silica nanoparticles (MSNs) have received increasing attention as an ideal carrier for drugs in clinical therapy.^[^ ^19^ ^]^ The MSN could be further used to deliver specific agents for sepsis therapy, aiming to avoid the complicated components in apoEVs and to enhance the therapeutic efficiency.

**Figure 4:**
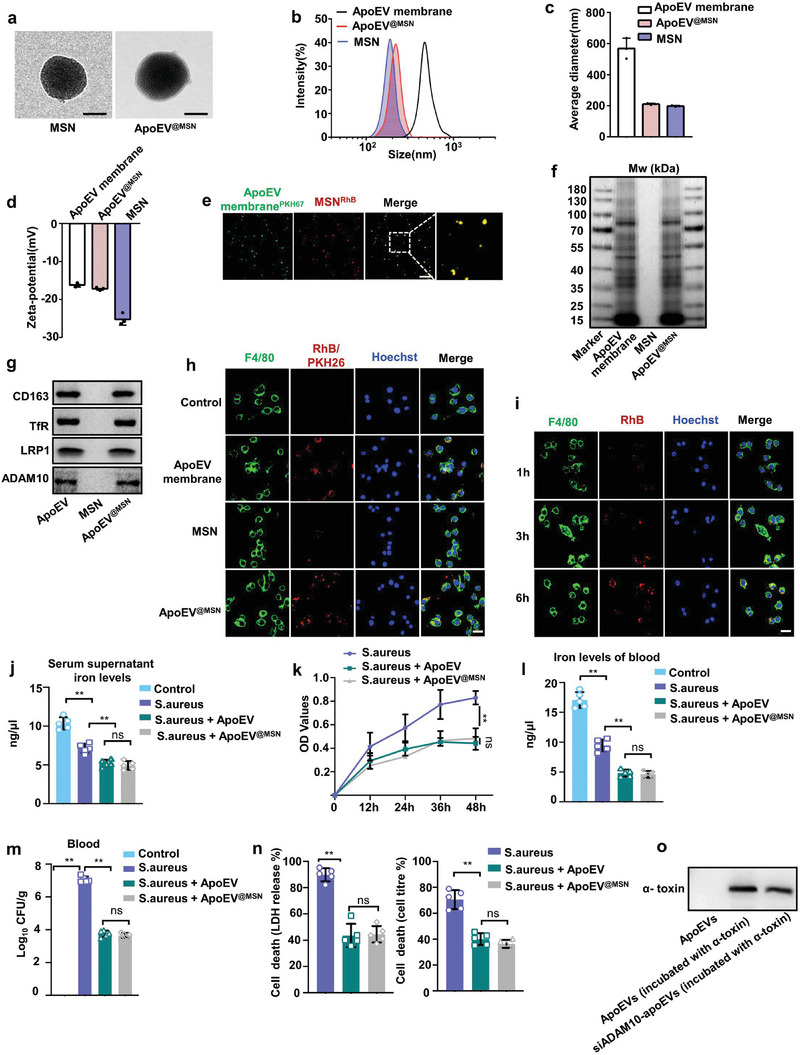
Construction and characterization of engineered apoEVs. a) Representative TEM image of MSN and apoEV^@MSN^. Scale bar, 100 nm. b) Size distribution of apoEVs membrane, MSNs, and apoEV^@MSN^ measured by DLS. c,d) Average diameter **c**), and surface zeta potential d) of apoEV membrane, MSNs, and apoEV^@MSN^. n = 3. e) Representative fluorescence images show the colocalization of MSNs and apoEVs membrane. Scale bars, 5 µm. f) Coomassie blue imaging of SDS‐PAGE protein analysis during apoEV^@MSN^ fabrication process. g) Western blot analysis for membrane‐specific protein markers. h) Representative fluorescence images show the uptake efficiency by macrophages. Scale bars, 20 µm. i) Time‐dependent uptake of apoEV^@MSN^ by macrophages. Scale bars, 20 µm. j) Total iron levels in the serum supernatant. n = 5. k) Growth curve of *S. aureus* in serum supernatant. n = 5. l) The iron level in serum after *S. aureus* infection. n = 5 mice. m) *S. aureus* burden in blood of infected mice receiving apoEVs or apoEV^@MSN^. n = 5 mice. n) Cell death following treatment with *S. aureus* together with apoEVs or apoEV^@MSN^. n = 5. o) Western blot analysis of α‐toxin neutralizing efficacy in apoEVs or apoEV^@MSN^. All results are representative of the data generated in at least three independent experiments. For (j–n) data are presented as means ± s.d., statistical significance was assessed by one‐way ANOVA with Tukey's post‐hoc test. ^*^ *p* < 0.05, ^**^ *p* < 0.01, ns, not significant.

TEM images directly showed the designed core‐shell structure of the apoEV^@MSN^ (**Figure** **4**a). Further information about the core‐shell structure formation of apoEV^@MSN^ was provided by DLS. The size distribution indicated the diameter of apoEV^@MSN^ increased ≈20 nm after the coating of apoEVs membrane on the MSN (Figure 4b,c). And zeta potential determination showed the coating of organic biological membrane improves the surface potential properties of inorganic nanoparticles (Figure 4d). The finished apoEV^@MSN^ was also visualized by confocal fluorescence microscopy, which exhibited a high colocalization degree of red MSNs and green apoEVs membrane (Figure 4e). The membrane integrity of apoEV^@MSN^ was verified by Coomassie blue staining to evaluate the overall protein profiles after apoEV^@MSN^ construction. The results indicated that the protein composition of apoEV^@MSN^ was quite similar to that of apoEVs membrane (Figure 4f). In addition, immunoblotting demonstrated the presence and enrichment of key surface antigens, including CD163, TfR, LRP1 and ADAM10 on apoEV^@MSN^, further confirming the translocation of apoEVs membrane onto the polymeric cores (Figure 4g). To investigate the cellular uptake of apoEV^@MSN^, we incubated macrophages with apoEVs membrane, MSNs, and apoEV^@MSN^. There was a significant red fluorescence signal in the apoEVs membrane and apoEV^@MSN^ groups compared to that in the control and MSN groups (Figure 4h), implying the high‐efficiency uptake of apoEV^@MSN^ by macrophages. Then, we examined the time‐dependent cell uptake of apoEV^@MSN^ by macrophages. As expected, the RhB fluorescence intensity increased with the incubation time (Figure 4i).

We further tested the iron binding capability of apoEV^@MSN^. It was determined using iron binding capacity test by the same experiment described above. Consistently, addition of apoEV^@MSN^ in *S. aureus*‐infected mouse serum induced a significant decrease in iron levels (Figure 4j) and *S. aureus* growth (Figure 4k). Furthermore, we also detected the iron binding capacity of these apoEV^@MSN^ in sepsis. Similarly, administration of apoEV^@MSN^ in vivo resulted in significant decrease in total iron levels (Figure 4l) and *S. aureus* growth (Figure 4m). We also evaluated whether these apoEV^@MSN^ could protect cells by binding toxins. Similarly, apoEV^@MSN^ protected target cells from α‐toxin as indicated either by LDH release or by metabolic activity (Figure 4n). Then we found apoEV@MSN can neutralize α‐toxin (Figure 4o). Collectively, these results provide strong evidence for the successful construction of engineered apoEVs (apoEV^@MSN^), which maintained the original morphology of natural apoEVs and exhibited a similar protein profile as that of natural apoEVs membrane, demonstrating that apoEV^@MSN^ inherited the structure and function of biomembranes.

### 2.5 Engineered ApoEVs Loaded with miR‐146a Attenuated Bacteria‐Induced Inflammation

The apoEVs membrane‐coated nanoparticles provide an alternative platform to harbor specific pharmaceutical content for further therapeutic potential. In the context of infection, accumulating evidence suggests that EVs play an important role in communication between the inflammatory microenvironments by transferring microRNAs (miRNAs) or other molecules.^[^ ^20^ ^]^ MicroRNA‐146a (miR‐146a), a widely reported negative immunoregulatory small noncoding RNA,^[^ ^21^ ^]^ was found to be one of the most enriched anti‐inflammatory miRNAs^[^ ^22^ ^]^ in apoEVs derived from *S. aureus*‐infected mice (Figure S3a, Supporting Information). Thus, engineered apoEVs‐based miR‐146a delivery was further accessed in sepsis therapy.

**Figure 5:**
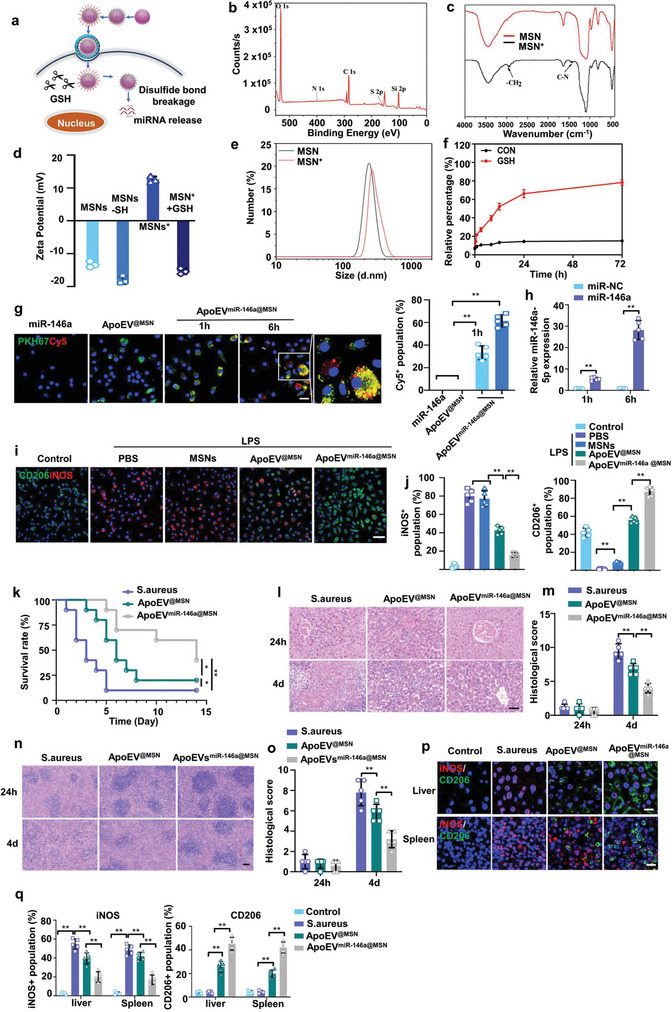
Engineered apoEVs loaded with miR‐146a attenuated the bacteria‐induced inflammation. a) Schematic diagram of delivery of miR‐146a from apoEV^miR‐146a@MSN^ and intracellular release of miR‐146a in the cytosol. b) X‐ray photoelectron spectroscopy (XPS) spectra of 2‐dimethylaminoethanethiol‐modified MSNs (MSNs‐DMAET). c) Infrared spectrometry of DMA‐grafted MSNs with disulfide bonds. d) Zeta potential of MSNs after each step of functionalization to form MSNs^+^. n = 3. e) Size distribution of MSNs and MSNs^+^. n = 3. f) The miR‐146a release profile from MSNs with or without GSH. n = 3. g) Time‐dependent uptake of miR‐146a (red) from apoEV^@MSN^ (green) by macrophages. Scale bars, 20 µm. h) Transfection efficiency of miR‐146a was determined by quantifying the miRNA level using real‐time quantitative PCR. n = 5. i,j) Representative fluorescence images of the macrophage phenotypes (i) and the percentage of the iNOS/CD206‐positive population(j). n = 5. Scale bars, 50 µm. k) Survival of uninfected mice, infected mice, infected mice receiving apoEV^@MSN^ or apoEV^miR‐146a@MSN^. n = 10 mice. l,m) H&E staining of representative liver sections l) and the associated histological scores m) at 24 h or 4 days after *S. aureus* infection. Scale bar, 50 µm. n = 5 mice. n,o) H&E staining of representative spleen sections n) and the associated histological scores o) at 24 h or 4 days after *S. aureus* infection. Scale bar, 50 µm. n = 5 mice. p,q) Representative fluorescence images of the macrophage phenotypes in liver or spleen p) and the percentage of the iNOS/CD206 positive population cells q) in each group. Scale bars, 50 µm. n = 5 mice. All results are representative of the data generated in at least three independent experiments. For (g,h,j,m,o,q) data are represented as the mean ± s.d., statistical significance was assessed by one‐way ANOVA with Tukey's post‐hoc test. For (k) statistical significance was assessed by the log‐rank test. ^*^ *p* < 0.05, ^**^ *p*  < 0.01.

Our design strategy is to trigger a responsive release of loaded miR‐146a after apoEV^@MSN^ enters the target cell. Specifically, the positive dimethylamine (DMA) group is first linked to MSNs by disulfide bonds (S─S bonds) to prepare positively charged MSNs (MSNs^+^), and miR‐146a is bonded to the surface of MSNs^+^ by electrostatic interaction to achieve the efficient loading of miRNA (Figure S3b, Supporting Information). Then miR‐146a@MSN was coated with the apoptotic membrane according to the above method to obtain apoEV^miR‐146a@MSN^ (Figure S3c, Supporting Information). When apoEV^miR‐146a@MSN^ enters the target cell, the S─S bond is broken by the high concentration of glutathione (GSH) in the cytoplasm, resulting in rapid release of miR‐146a to participate in downstream cell function regulation (**Figure** **5**a). The miRNA encapsulation efficiency of MSNs at different cargo/MSN weight ratios are listed in Table S1 (Supporting Information). The encapsulation efficiency of miRNA reached 99%, which demonstrated the excellent loading capacity of MSNs. The chemical components of apoEV^miR‐146a@MSN^ were determined by X‐ray photoelectron spectroscopy (XPS), which showed obvious signals of nitrogen and sulfur, indicating the successful grafting of DMA on MSNs with S─S bonds (Figure 5b). The existence of carbon‐nitrogen bonds (C─N) in the DMA group was also confirmed by the infrared spectrometry (Figure 5c). During the fabrication process, the surface properties of the product change in accordance with the electronic properties of certain function groups, and finally form MSN^+^ which can be broken by GSH‐mediated cleavage of the S─S bonds (Figure 5d). At the same time, the introduction of DMA group did not obviously affect the particle size of MSN (Figure 5e). To demonstrate the GSH‐triggered release of miR‐146a, miR‐146a@MSNs were incubated in PBS solution and GSH solution for 3 days. The results showed that GSH triggered a responsive sustained release of miR‐146a, which could not be achieved in PBS (Figure 5f). After characterizing the formation and function of the miR‐146a–loaded MSNs, they were further fused with apoEVs membranes to produce apoEV^miR‐146a@MSN^.

Next, the biological properties of apoEV^miR‐146a@MSN^ were studied at the cellular level. The transfection efficiency was determined by quantifying the fluorescence intensity (Figure 5g) and the miRNA expression level (Figure 5h). As shown in Figure 5g, apoEVs^miR‐146a@MSN^ could effectively carry Cy5 labeled miR‐146a into cell, overcoming the dilemma that free miR‐146a was unable to enter the cell alone. And with the action of GSH, miR‐146a is gradually dissociated from apoEVs^miR‐146a@MSN^ and distributed throughout the cytoplasm. The inflammatory regulation ability of apoEVs^miR‐146a@MSN^ was further evaluated by incubation with the inflammatory macrophages in vitro. LPS was added to BMDMs to induce inflammation before the administration of MSNs, apoEV^@MSN^ or apoEV^miR‐146a@MSN^. We examined whether miR‐146a delivered by engineered apoEVs could exert anti‐inflammation by regulating the M1 and M2 activation states (Figure 5i). Immunofluorescence analysis revealed apoEV^miR‐146a@MSN^ attenuated the LPS‐induced increase in iNOS expression (Figure 5i,j). As a M2 activation state marker, CD206 was significantly enhanced in apoEV^miR‐146a@MSN^ group (Figure 5i,j). Western blot analysis demonstrated similar findings to immunofluorescence analysis (Figure S4a, Supporting Information). Enzyme‐linked immunosorbent assay (ELISA) showed that apoEV^miR‐146a@MSN^ distinctly reduced the levels of proinflammatory factors (TNF‐α and IL‐1β) (Figure S4b, Supporting Information). To further evaluate efficacy of apoEV^miR‐146a@MSN^ to ameliorate inflammation in vivo, apoEV^miR‐146a@MSN^ was injected into *S. aureus* induced sepsis model. Alleviated concentrations of proinflammatory factors TNF‐α, IL‐1β in serum were observed for the apoEV^miR‐146a@MSN^ group as compared to the *S. aureus* group or apoEV^@MSN^ group (Figure S4c, Supporting Information). Consistently, administration of apoEV^miR‐146a@MSN^ extended the survival (Figure 5k) and ameliorated tissue injury of *S. aureus*‐infected septic mice (Figure 5l–o). Mechanistically, apoEV^miR‐146a@MSN^ attenuated tissue injury by converting macrophages to an anti‐inflammatory phenotype to resolve local inflammation, which was marked by a significant increase in the CD206‐positive staining rate and a reduction in the iNOS‐positive staining rate (Figure 5p,q).

Collectively, apoEV^miR‐146a@MSN^ possesses an antigenic exterior identical to natural apoEVs membrane, thus inheriting their capability to restrict iron accessibility and bind to toxins. In addition, apoEV^miR‐146a@MSN^ acts as inhibitor of inflammation. These two functionalities together enable effective intervention during inflammatory hyperactivation, providing a promising therapeutic intervention for the treatment of sepsis.

### 2.6 Red Blood Cell‐Hitchhiking Boosted the Therapeutic Efficacy of ApoEV^miR‐146a@MSN^

As demonstrated above, apoEV^miR‐146a@MSN^ possessed many potential advantageous features in terms of their intrinsic therapeutic properties and ability to deliver functional cargo. However, since the infused apoEV^miR‐146a@MSN^ have no propulsive forces, they are restricted in viscous blood fluid and suffer from rapid clearance by phagocytes, leading to compromised bioavailability especially in severe infection. To determine the biodistribution of apoEVs^miR‐146a@MSN^ after injection, DiD‐labeled apoEVs were intravenously injected into mice. Ex vivo fluorescent imaging revealed that most of the infused apoEV^miR‐146a@MSN^ homed to the liver or spleen within 1 h (Figure S5a, Supporting Information). Meanwhile, confocal images revealed that the infused engineered apoEVs were mainly taken up in the livers or spleens (Figure S5b, Supporting Information). Then, we employed the strategy to leverage red blood cells (RBCs) “hitch‐hiking”, which could not only prolong the circulating time of apoEV^miR‐146a@MSN^, but also enhance their contact with iron and toxins in viscous flow, aiming to enhance their ability to capture iron‐containing proteins and to neutralize toxins.

**Figure 6:**
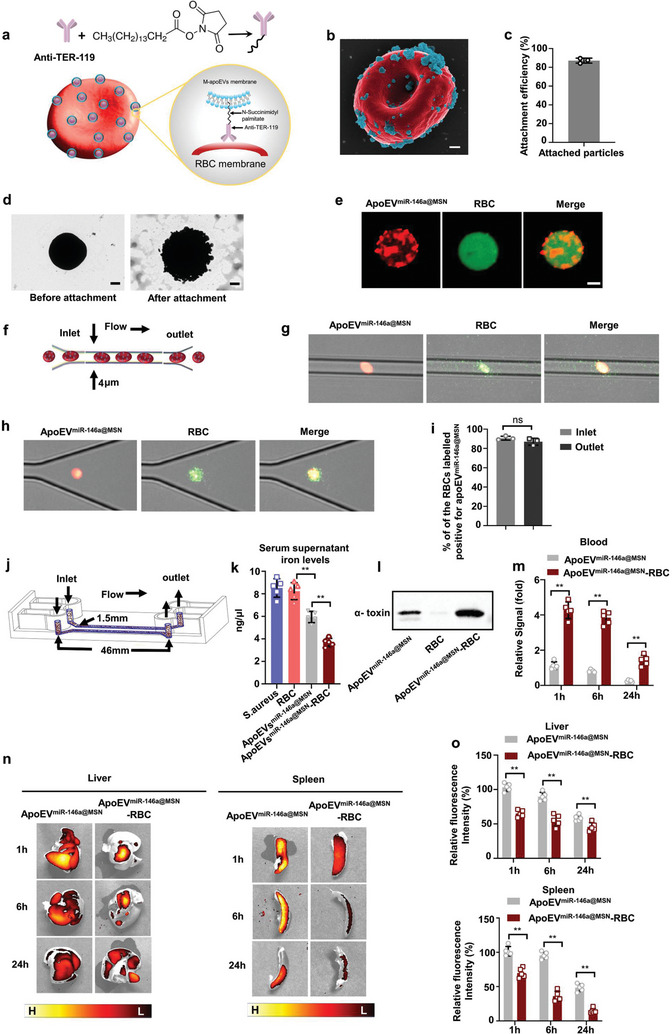
Red blood cell‐hitchhiking boosts the therapeutic efficacy of apoEV^miR‐146a@MSN^. a) Schematic of the attachment of apoEV^miR‐146a@MSN^ to RBCs through TER119‐PN binding complex. b) Scanning electron micrographs (pseudocolored red, RBC; pseudocolored green, apoEV^miR‐146a@MSN^) of an example apoEV^miR‐146a@MSN^‐RBC. Scale bar: 400 nm. c) Attachment efficiencies of apoEV^miR‐146a@MSN^ onto RBCs. n = 3. d) Transmission electron micrograph of RBC before or after apoEVs^miR‐146a@MSN^ attachment. Scale bar: 1 µm. e) Fluorescent images of apoEV^miR‐146a@MSN^‐RBC. ApoEV^miR‐146a@MSN^ were labelled using Did (orange red) and the RBCs were labelled using 5‐(Octadecanoylamino) (green). Scale bar: 2 µm f) Schematics of the microfluidic chip. g) Fluorescently labeled apoEV^miR‐146a@MSN^‐RBC were able to squeeze with ease through microchannels with a width of 4 µm, which is smaller than the size of RBCs (5–6 µm). h) RBCs preserved their integrity when collected at the outlet. i) Change in percentage of apoEV^miR‐146a@MSN^‐RBC after squeezing through microfluidic channels was not statistically. j) Schematics of the flow chip. The flow channel is 46 mm long and 1.5 mm wide. k) Total iron levels in the serum supernatant. n = 5. l) Western blot analysis of α‐toxin neutralizing efficacy in unmodified apoEV^miR‐146a@MSN^ or apoEV^miR‐146a@MSN^‐RBC. n = 5. m) In vivo circulation time of unmodified apoEV^miR‐146a@MSN^ or apoEV^miR‐146a@MSN^‐RBC. n = 5 mice. n) Ex vivo fluorescent imaging of livers or spleen at various timepoints after systemic administration with unmodified apoEV^miR‐146a@MSN^ or apoEV^miR‐146a@MSN^‐RBC. o) Comparison of the relative fluorescence intensities for unmodified apoEV^miR‐146a@MSN^ or apoEV^miR‐146a@MSN^‐RBC in liver or spleen. n = 5 mice. All results are representative of the data generated in at least three independent experiments. For (i,m) data are represented as the mean ± s.d., statistical significance was assessed by unpaired two‐tailed Student's t test. For (k,o) data are represented as the mean ± s.d., statistical significance was assessed by one‐way ANOVA with Tukey's post‐hoc test. ^*^ *p* < 0.05, ^**^ *p* < 0.01, ns, not significant.

Construction of the RBCs driven apoEV^miR‐146a@MSN^ was realized by the attachment of apoEVs^miR‐146a@MSN^, to RBCs through TER119 antibody‐Palmitic acid N‐hydroxysuccinimide ester (PN) binding complex (PN binds to the amino groups of TER119 through the NHS end)^[^ ^23^ ^]^ (**Figure** **6**a). Figure 6b shows an electron micrograph of apoEV^miR‐146a@MSN^ attached on RBCs. Figure 6c shows attachment efficiency of apoEV^miR‐146a@MSN^ to the surface of RBCs. Figure 6d shows transmission electron micrograph of RBC before or after apoEV^miR‐146a@MSN^ attachment. The completeness of the apoEV^miR‐146a@MSN^‐RBC was also investigated by confocal laser scanning microscopy, and the red apoEV^miR‐146a@MSN^ and green RBC exhibited a high degree of colocalization, indicating the successful association of the two components (Figure 6e). MALDI‐TOF mass spectrometry showed that PN could be anchored into TER119 via the cross‐linking between N‐hydroxy succinimide ester and NH2‐group of TER119 (Figure S6, Supporting Information).

Inside the body, motility and transportation of RBCs occur through deformation and adaptation of the cells to tissue microenvironment. Therefore, the ability to deform and pass through confined spaces without losing the cargo is critical for in vivo operation. To investigate deformability of the proposed apoEV^miR‐146a@MSN^‐RBC and stability of their components, we injected and squeezed them through microfluidic channels with a width of 4 µm, which is smaller than the size of the RBCs (5–6 µm) (Figure 6f). ApoEV^miR‐146a@MSN^‐RBC were able to deform inside and pass through the microchannels (Figure 6g). Moreover, the apoEV^miR‐146a@MSN^‐RBC preserved their integrity when deformed inside the microchannels and collected at the outlet (Figure 6h). The flow cytometric analysis revealed that 90.7% of the RBC population labeled positive for apoEV^miR‐146a@MSN^ before injection. The percentage did not decrease significantly after squeezing through the microchannels (≈87%) (Figure 6i).

We hypothesized that the flow of RBCs through the circulation could highly improve microcosmic moving ability of the attached engineered apoEVs. To explore whether RBC‐hitchhiking could improve the therapeutic efficacy of apoEV^miR‐146a@MSN^, we modeled blood flow in artificial microfluidic devices (Figure 6j; Figure S7, Supporting Information). The infected mice‐derived serum combined with unmodified apoEV^miR‐146a@MSN^, RBCs or apoEV^miR‐146a@MSN^‐RBC was injected into the tube directly plugged into the inlet via Teflon tubing. The samples were collected at the outlet of the chip. The infused apoEV^miR‐146a@MSN^ or RBCs were isolated by centrifugation for toxin analysis and serum supernatant was collected for iron amount analysis. The results showed that the attachment of RBCs enhanced iron binding (Figure 6k) and α‐toxin neutralizing (Figure 6l), indicating RBC‐hitchhiking boosts the therapeutic efficacy of apoEV^miR‐146a@MSN^. The greatest advantage of RBC‐hitchhiking is their ability to achieve a prolonged circulatory effect in the biological environment in vivo. The retention of apoEV^miR‐146a@MSN^ in the circulation was detected by fluorescence intensity at various time points (Figure 6m). The blood level of apoEVs^miR‐146a@MSN^‐RBC was ≈3–5 times higher than that of free apoEV^miR‐146a@MSN^ at all‐time points. Therefore, as expected, RBC carrier markedly prolongs apoEV^miR‐146a@MSN^ circulation. The distribution of apoEV^miR‐146a@MSN^‐RBC was further examined after systemic administration (Figure 6n). Leveraging fluorescence from the apoEVs membrane, and the distribution was visualized by ex vivo fluorescence imaging of the excised tissues. Fluorescence from unmodified apoEV^miR‐146a@MSN^ permeated throughout the liver or spleen tissue within 1 h, and the strong signal was retained for at least 24 h. In contrast, apoEV^miR‐146a@MSN^‐RBC exhibited relatively low accumulation in liver or spleen over the course of 24 h. The normalized fluorescence data also verified the lower accumulation of RBC‐driven apoEV^miR‐146a@MSN^ in liver /spleen compared with unmodified apoEV^miR‐146a@MSN^ (Figure 6o). Overall, these data indicate that the motion behavior of apoEV^miR‐146a@MSN^ driven by RBCs greatly decreased liver and spleen clearance and improved their therapeutic effects.

### 2.7 RBC Hitchhiking ApoEV^miR‐146a@MSN^ Ameliorated Severe Sepsis

Sepsis patients with higher iron levels are associated with an increase in mortality and become a huge challenge for clinical treatment.^[^ ^24^ ^]^ Having showed that engineered apoEVs could bind iron or iron‐containing proteins, then serve as circulating “iron catchers” to prevent bacteria from iron acquisition. In addition, it was found that RBC hitchhiking significantly enhanced the retention of engineered apoEVs in vivo. Therefore, we hypothesized that the efficient enhanced host iron recycling by RBC hitchhiking can contribute to the survival of septic models with high serum iron levels.

**Figure 7:**
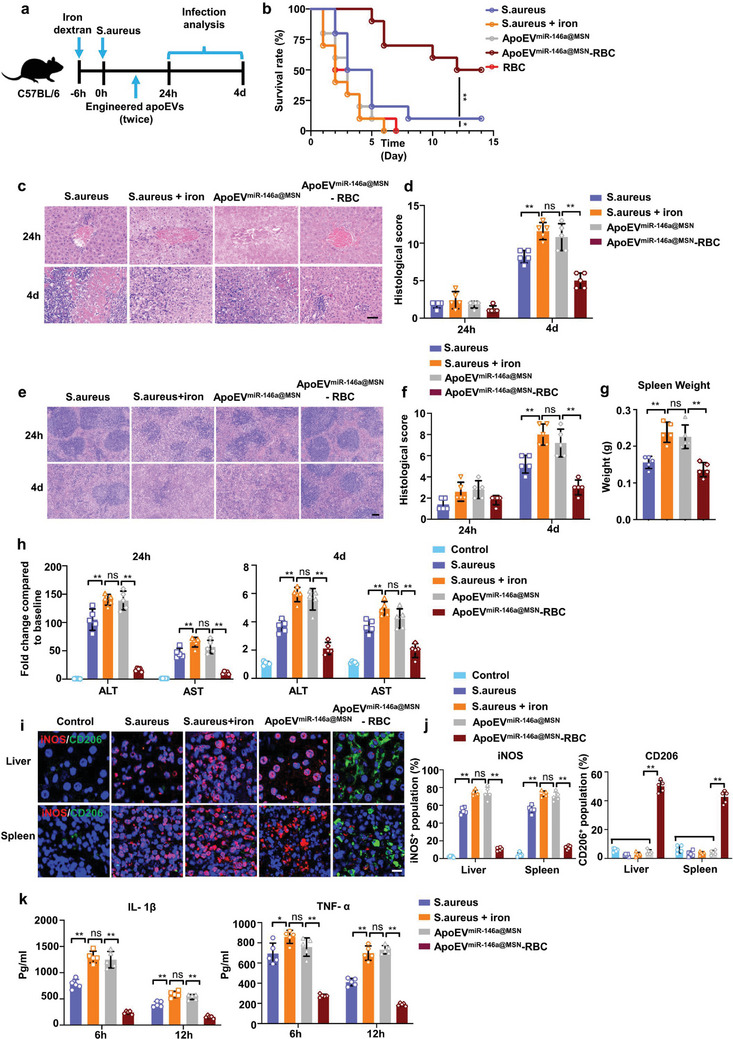
RBC hitchhiking ameliorates a severe disease model. a) Schematic diagram of the experimental procedure. Iron‐loaded mice were injected with engineered apoEVs at 1 or 6 h after *S. aureus* infection. b) Survival of iron‐loaded mice receiving unmodified apoEV^miR‐146a@MSN^ or apoEV^miR‐146a@MSN^‐RBC. n = 10 mice. c, d)H&E staining of representative liver sections c) and the associated histological scores d) at 24 hours or 4 days after engineered apoEVs injection. Scale bar, 50 µm. n = 5 mice. e,f) H&E staining of representative spleen sections e) and the associated histological scores f) at 24 h or 4 days after engineered apoEVs injection. Scale bar, 50 µm. n = 5 mice. g) The spleen weight at 4 days after engineered apoEVs injection. n = 5 mice. h) ALT and AST levels in the serum of iron‐loaded mice receiving unmodified apoEV^miR‐146a@MSN^ or apoEV^miR‐146a@MSN^‐RBC. n = 5 mice. i) Representative fluorescence images of the macrophage phenotypes in liver or spleen. Scale bars, 20 µm. j) Quantitative analysis of the iNOS^+^/CD206^+^ cells in each group. n = 5 mice. k) Concentration profiles of TNF‐α and IL‐1β in the serum of iron‐loaded mice treated with different groups. n = 5 mice. All results are representative of the data generated in at least three independent experiments. For (d,f,g,h,j,k) data are represented as the mean ± s.d. For (d,g,h,j,k) statistical significance was assessed by one‐way ANOVA with Tukey's post‐hoc test. For (f) statistical significance was assessed by Kruskal–Wallis test. For (b), statistical significance was assessed by the log‐rank test. ^*^ *p* < 0.05, ^**^ *p* < 0.01, ns, not significant.

We first examined the survival and histological changes in iron overload‐infected mice (**Figure** **7**a). The results showed that, compared to *S. aureus*‐infected mice, iron‐dextran treatment induced shortened survival (Figure 7b) and more extensive necrosis in the liver (Figure 7c), disturbed architecture in the spleen (Figure 7e), elevated histological injury score of liver and spleen (Figure 7d,f), and aggravated splenomegaly (Figure 7g). In the study, apoEV^miR‐146a@MSN−^RBC or unmodified apoEV^miR‐146a@MSN^ were injected into the iron overload infection model. The results showed that, administration of unmodified engineered apoEVs did not significantly improve survival or histological changes in iron overload infected mice (Figure 7b–g). While administration of apoEV^miR‐146a@MSN^‐RBC greatly extended the survival of septic mice with high serum iron level (Figure 7b). Histological analysis revealed that the iron overload infected mice treated with apoEVs^miR‐146a@MSN^‐RBC exhibited less severe tissue injuries (Figure 7c,e), decreased histological scores (Figure 7d,f) and alleviated spleen enlargement (Figure 7g), indicating RBC hitchhiking improved the efficacy of engineered apoEVs therapy. In addition, the apoEVs^miR‐146a@MSN^‐RBC treatment significantly attenuated the enhanced serum ALT and AST levels in the iron overload infected mice (Figure 7h). Mechanistically, the fluorescence images showed that the administration of apoEVs^miR‐146a@MSN^‐RBC further promoted the conversion of macrophages to the M2 phenotype in favor of tissue homeostasis (Figure 7i,j). To examine the potential systemic response, we monitored the serum levels of TNF‐α and IL‐1β in iron overload‐infected mice. Indeed, alleviated concentrations of these cytokines were observed for the apoEV^miR‐146a@MSN^‐RBC treatment as compared to the unmodified apoEV^miR‐146a@MSN^ group, indicating an effective reduction of inflammation at the systemic level (Figure 7k). Thus, these experiments are proof of principle that RBC hitchhiking can strongly augment the therapeutic efficiency of engineered apoEVs and thereby ameliorate a severe sepsis model.

### 2.8 Biocompatibility of Engineered ApoEVs

To assess the biosafety of engineered apoEVs in vivo, the weights and blood of mice were analyzed after administration of apoEV^miR‐146a@MSN^ or apoEV^miR‐146a@MSN^‐RBC. As shown in Figure S8a (Supporting Information), no obvious reduction was observed in the body weight of apoEV^miR‐146a@MSN^ or apoEV^miR‐146a@MSN^‐RBC treated mice over time. Meanwhile, the proportions of red blood cells (RBCs), white blood Cells (WBCs) or platelets (PLTs) exhibited no significant differences between the different groups (Figure S8b, Supporting Information). The functional indicators of RBCs (HGB, MCV, MCH, and MCHC) also showed no significant differences between PBS group and the treated groups (Figure S8c, Supporting Information). Moreover, the safety of engineered apoEVs was evaluated by performing a serum biochemical test. According to the results, treatment of apoEV^miR‐146a@MSN^ or apoEV^miR‐146a@MSN^‐RBC did not lead to significant changes in hepatorenal function (Figure S8d, Supporting Information). Histological examination revealed no noticeable acute organ damage after engineered apoEVs treatments, suggesting the lack of in vivo toxicity caused by engineered apoEVs (Figure S8e, Supporting Information). In addition, the cytotoxicity test demonstrated that engineered apoEVs had no obvious cytotoxic effects on macrophages and A549 cells (Figure S8f, Supporting Information). Collectively, these results provide strong evidence about the excellent biocompatibility of engineered apoEVs platform for delivery of therapeutics.

## 3 Discussion

The host's programmed cell death pathways, including pyroptosis and necroptosis, have been extensively studied in the context of infection,^[^ ^25^, ^26^ ^]^ whereas the role of apoptosis in infection is much less studied. Recent studies have shown that promoting macrophages apoptosis in sepsis could lead to improved outcomes, including increased survival, reduced local and systemic inflammation, reduced tissue injury, and inhibited bacterial dissemination in the blood.^[^ ^27^ ^]^ In our study, we found that during *S. aureus* infection, macrophage apoptosis was triggered, resulting in the release of apoEVs into circulation. These apoEVs played a beneficial role by acting as decoys to neutralize bacterial toxins and bind iron, thus promoting iron sequestration and preventing their detrimental effects. This previously unknown humoral regulation mechanism highlights the potential of apoEVs as a therapeutic tool for sepsis.

Apoptosis is a well‐characterized process that leads to the production of apoEVs.^[^ ^8^, ^28^ ^]^ While most of the studies on EVs in bacterial infection have focused on the mechanisms for antigen delivering or signal transduction,^[^ ^29^ ^]^ other biological functions of apoEVs still need to be explored. Our findings suggest that increased release of macrophages‐derived apoEVs during bacterial infection serve as effective toxin neutralizers and iron recyclers. In this study, we found that TfR, CD163, LRP1 and ADAM10 are integrated on apoEVs surface, which ensures the toxin neutralization and iron recycling capacity of apoEVs to fight against infections. These toxin and iron catchers enhance the resistance to bacterial infection, thereby highlighting a previously unknown mechanism of innate immunity.

Despite the potential therapeutic benefits of natural apoEVs, their heterogeneity and complex composition pose challenges in terms of efficacy and safety concerns.^[^ ^30^, ^31^ ^]^ Moreover, issues such as efficient isolation, scale‐up production of apoEVs and accurate monitoring of the dosage of therapeutic cargo needs to be addressed for clinical applications.^[^ ^30^ ^]^ Thus, engineered EVs have recently emerged as a promising alternative approach for EVs‐based therapy,^[^ ^32^ ^]^ offering the advantage of tailoring their properties to specific therapeutic needs. EVs membrane‐coated nanoparticles (NPs) have recently emerged as attractive nanomedical tools owing to their EVs surface mimetic features and tailored nanomaterial characteristics.^[^ ^33^ ^]^ In addition, miR‐146a has been shown to be effective at managing the inflammation associated with sepsis.^[^ ^34^ ^]^ The modification of apoEVs allows for the delivery of therapeutic cargo, further boosting its therapeutic effect.

With multiple functions, miR‐146a was reported to regulate inflammation via NF‐κB signaling pathway.^[^ ^22a^ ^]^ It is shown that miR‐146a, induced by macrophage‐derived inflammatory signals, suppresses inflammatory reaction in both adipose and periodontal tissues.^[^ ^35^ ^]^ In addition, it is reported that miR‐146a plays a role in the regulation of inflammation in orbital fibroblasts.^[^ ^36^ ^]^ In this study, miR‐146a was found to be one of the most enriched miRNAs in apoEVs‐derived from *S. aureus*‐infected mice as indicated by miRNA sequencing experiment. We demonstrated that engineered apoEVs‐based miR‐146a delivery reduced multiple proinflammatory factors, resulting in ameliorated tissue injury of *S. aureus*‐infected septic mice.

It is well established that the movement of bacteria in a viscous “blood fluid” is driven by the rotation of flagella which provides active propulsion.^[^ ^37^ ^]^ However, nano‐sized particles lack propulsive forces and their mobility is often reduced when diffusing in viscous flow. The therapeutic efficiency of nano‐sized engineered apoEVs relies on efficient contact with the target molecules in the circulation. Therefore, it is reasonable to hypothesize that addition of active forces may increase the therapeutic efficiency of engineered apoEVs.^[^ ^38^ ^]^ Most of the studies on engineered EVs‐based therapy have focused on the in vivo distribution, retention, and clearance of EVs in the circulation, the micromotion of nano‐sized engineered EVs is rarely considered. In our study, we employed mesoporous silica nanoparticles (MSNs) preloaded with anti‐inflammatory agents (microRNA‐146a) to modify the apoEVs, thereby enhancing their therapeutic profile. Moreover, to address the limited mobility of nano‐sized particles in the highly viscous biological fluids, we leveraged the natural movement of red blood cells (RBCs) to enhance the microcosmic moving ability by tethering the engineered apoEVs to them. The rotation and deformation of RBCs allow for the propulsion of engineered apoEVs, thereby increasing the contact efficiency with the target molecule. This innovative approach, referred to as apoEV^miR‐146a@MSN^‐RBC demonstrates improved therapeutic efficacy in an iron overload infection murine model. According to previous studies, iron dextran is phagocytosed within the reticuloendothelial system and ferrous iron is then released into blood.^[^ ^39^ ^]^ Once in the blood, ferrous iron is immediately oxidized to ferric iron.^[^ ^39b^ ^]^ Ferric iron bind to transferrin to increase transferrin saturation^[^ ^40^ ^]^ and can be used for hemoglobin synthesis, which leads to an increase in hemoglobin concentration.^[^ ^41^ ^]^ These results highlight that RBC hitchhiking can strongly augment the therapeutic efficiency of engineered apoEVs, allowing RBC‐based propulsive forces to be employed as a novel platform for active engineered EVs delivery to treat severe disease.

There were some limitations in our study. We mainly focused on the apoEVs derived from macrophages, the roles of apoEVs from other cellular origins in sepsis remains to be determined. It has been shown that the *S. aureus* infection also induce apoptosis of other kinds of cells including T cells, B cells, neutrophils, epithelial cells, endothelial cells, fibroblasts and keratinocytes.^[^ ^42^ ^]^ The outcome of apoptosis inhibition of these cells during *S. aureus* infection may depend on the production from the apoptotic cells, and need to be further investigated. Additionally, evaluation of other small molecules present in the apoEVs membranes that may also participate in inhibition of sepsis progression would be valuable. Future studies may provide additional evidence for apoEVs‐based therapy for sepsis.

In summary, our study presents a proof of concept for engineered apoEVs (i.e., the apoEV^miR‐146a@MSN^‐RBC) with multifunctionality including i) bacterial toxin neutralization, ii) iron sequestration, iii) anti‐inflammation and iv) RBC‐driven propulsive forces. The synergy among these functions demonstrated significant therapeutic potential in the treatment of severe sepsis as evidenced by extended survival, reduced inflammation, and attenuated tissue injury. This study established a technological platform of engineered apoEVs and paved the way for further exploration of their potential in sepsis treatment and the delivery of therapeutic agents to treat other kinds of bacterial infections.

## 4 Experimental Section

### Animal Care

Animal protocols related to this study were reviewed and approved by the Institutional Animal Care and Use Committee at the School of Medicine of Shanghai Jiao Tong University. All experiments were performed in accordance with the guidelines published by the Institutional Animal Care and Use Committee at the School of Medicine of Shanghai Jiao Tong University, Shanghai.

### Bacterial Strains and Growth Conditions

*Staphylococcus aureus* (*S. aureus* Newman, ATCC 13420) was purchased from American Type Culture Collection. *S. aureus* was grown overnight in Luria‐Bertani (LB) broth (10 g tryptone, 5 g yeast extract, 10 g NaCl per liter) at 37 °C with shaking. Bacteria density was confirmed by dilution plating.

### Animal Experiments

Six to eight week‐old female C57BL/6J were obtained from the Ninth People's Hospital Animal Center (Shanghai, China). Mice were intravenously treated with 3 × 10^7^CFU of *S. aureus* to induce sepsis. In addition, mice were intravenously injected with iron‐dextran (50 µg g^−1^ body weight, Sigma) to establish iron‐loaded mouse model.

To identify the role of apoptosis of macrophage in sepsis, liposome‐encapsulated Z‐VAD‐FMK was prepared with hydrogenated soybean phosphatidylcholine (Sinopharm) and cholesterol (Sinopharm) by the ethanol injection method.^[^ ^43^ ^]^ Mice were injected with liposome‐encapsulated Z‐VAD‐FMK (10 mg k^−1^g body weight) or equivalent volume of empty liposome at 12 h before infection. To identify the function of apoEVs or engineered apoEVs in *S. aureus* infection, mice were intravenously injected with apoEVs (50 µg of membrane protein) or engineered apoEVs (50 µg of membrane protein) at 1 and 6 h after *S. aureus* infection. Mice were euthanized at 24 h or 4 days after infection for histopathological evaluation. The survival rate of the mice was monitored every 12 h for 14 days. To test the changes in iron, blood was collected at 12 h after *S. aureus* infection.

### Cell Culture

Bone marrow‐derived macrophages (BMDMs) were obtained by harvesting bone marrow from C57BL/6J mice as previously described.^[^ ^44^ ^]^ Briefly, bone marrow cells were flushed out with PBS and lysed with red blood cell lysing buffer (Beyotime). After centrifuging for 5 min at 800 × g, the cells were seeded in plates and incubated with RPMI 1640 medium containing 10% fetal bovine serum (FBS) (Gibco, Gaithersburg, USA) and 20 ng mL^−1^ macrophage colony‐stimulating factor (M‐CSF) (PeproTech). Mature BMDMs were used for the next experiments for 7–8 days. The human lung epithelial cell line, A549, was purchased from American Type Culture Collection (ATCC, CCL‐185) and maintained in DMEM (Gibco, USA) supplemented with 10% FBS and 1% penicillin/streptomycin.

### Cytotoxicity Assay

The protective ability of apoEVs on cell death was determined as previously described.^[^ ^5a^ ^]^ Briefly, 3 × 10^4^ A549 cells were seeded in 96‐well plates and allowed to attach overnight. α‐toxin (H9395, Sigma–Aldrich, USA) was then added and incubated together for 3 h at 37 °C. The apoEVs or engineered apoEVs were mixed with α‐toxin in PBS. The apoEVs/α‐toxin or apoEV^@MSN^/α‐toxin mixture was incubated at 37 °C for 30 min and then added to plated A549 cells. The concentration of α‐toxin in the above experiments was 1 µg mL^−1^. Cell viability was determined either by LDH release (which indicates pore formation; Promega CytoTox‐One Kit, Promega, USA) or by metabolic activity via CellTiter (Promega, USA). Total cytolysis was calculated according to the manufacturer's instructions.

### The Immunoregulatory Effect of the Apoptotic Membrane In Vitro

To explore the immunoregulatory effect mediated by the engineered apoEVs, macrophages were treated with LPS (1 µg mL^−1^) to induce inflammation. Simultaneously, MSNs, apoEVs^@MSN^, or apoEVs^miR‐146a@MSN^ (30 µg mL^−1^ apoEVs‐membrane protein) were added to the culture system, and PBS and unstimulated macrophages were prepared in parallel for comparison. After 24 h, the cells were fixed with 4% PFA at 4 °C, followed by blocking with normal goat serum. Subsequently, the cells were incubated with anti‐CD206 antibody (ab64693, abcam, USA) or anti‐iNOS antibody (18985‐1‐AP, proteintech, USA) at 4 °C overnight and stained with hoechst33342 (Selleck Chemicals, USA). The fluorescence imaging was observed by confocal laser scanning microscopy (CLSM) (Nikon, Japan). The protein level was analyzed by western blotting. The detected proteins included iNOS (#13120, CST, USA) and CD206 (#24595, CST, USA), and GAPDH (CW0100, CwBio, China) was used as the internal reference. The levels of TNF‐α, IL‐1β in cell culture supernatants were detected with ELISA kits according to the instructions (Novus, Biologicals, China). For the in vivo measurements, the tissues were harvested to prepare frozen sections. After fixation and permeation with 0.1% Triton X‐100 (RT, 10 min), the cells or tissues were blocked with normal goat serum (37 °C, 1 h) and stained with iNOS (18985‐1‐AP, proteintech, USA) or CD206 (141703, Biolegend, USA) at 4 °C overnight, followed by counterstaining with hoechst33342 for 15 min. Fluorescence imaging was performed with confocal microscopy. The levels of TNF‐α and IL‐1β in the blood were determined using an ELISA kit according to the manufacturer's recommended protocol (Novus, Biologicals, China).

### SiRNA and Transfection

BMDMs were transfected with siRNA (Ribobio, China) targeting TfR or ADAM10 mRNAs using the advanced RNA Transfection Reagent (Zeta life, USA) according to the manufacturer's instruction. Subsequent treatments on transfected cells were performed 24 h post‐transfection.

### α‐Toxin Neutralization Assay

The apoEV (150 µg apoEVs‐membrane protein) or engineered apoEVs (150 µg apoEVs‐membrane protein) were resuspended in PBS respectively. α‐toxin was added to apoEVs suspension at a concentration of 1 µg mL^−1^. Following incubation, the apoEVs/α‐toxin or apoEV^@MSN^/α‐toxin mixture was resuspended in 40 mL PBS and spun at 2000 g for 20 min to pellet apoEVs or apoEV^@MSN^ with bound α‐toxin and remove excess α‐toxin. The α‐toxin neutralized by apoEVs or apoEV^@MSN^ were examined by western blotting.

### Serum Iron Measurements

According to the procedure previously described,^[^ ^45^ ^]^ blood was collected into EP tubes and allowed to clot at 4 °C for 12 h followed by centrifugation at 2500 rpm for 10 min. The serum was used for iron level analysis. For iron‐binding assay in vitro, the apoEVs or engineered apoEVs were added to the serum. After incubation, the added apoEVs or engineered apoEVs were isolated by centrifugation at 2000 g for 20 min at 4 °C. The serum supernatant and the apoEVs fraction were collected respectively. The total iron was detected using an Iron Assay Kit (abcam, USA) according to the manufacturer's instructions and was measured at 593 nm using microplate reader (Bio‐Rad, USA). To detect the bacteria growth in serum, the serum supernatant was heated at 56 °C for 1 h and *S. aureus* was inoculated into the supernatant. The cultures were then maintained under continuous shaking at 37 °C with optical measurements at OD490 or OD620 every 12 h.

### The Determination of Bacteria Burden in Blood

The blood was collected in the tube with heparin sodium to prevent blood from coagulation. Bacteria CFU were counted by plating dilutions of blood on LB plates after incubation at 37 °C for 12 h.

### ApoEVs Isolation and Characterization

BMDMs were treated with staurosporine (1 µm, HY‐15141, MCE, USA) for 3–4 h to induce apoptosis. Then, the culture media were collected and centrifuged at 100 g for 5 min to remove the cells and debris. After repeating this twice, the supernatant was further centrifuged at 2000 g (20 min) to concentrate the apoEVs in the pellet. Then, the pellet was suspended with PBS and used for subsequent experiments. The protein concentration was determined using a BCA protein assay kit (Thermo Scientific, USA). DLS analysis was performed using a Zetasizer Nano ZSE (Malvern, UK). The morphology of the apoEVs was observed by SEM (Hitachi, Japan). Annexin V‐FITC staining was conducted using an Annexin V‐FITC/PI apoptosis assay kit (A005‐2, 7Sea Biotech, China). ApoEVs were incubated with anti‐mouse C1q antibody (Cedarlane, CL7501F, USA) at 37 °C for 30 min. Then, fluorescence imaging was conducted with CLSM (Nikon, Japan).

Surface markers on BMDMs, apoptotic BMDMs, and apoEVs were examined by western blotting. The protein samples from macrophages, apoptotic macrophages, and apoEVs were loaded into the Bio‐Rad Electrophoresis System. The proteins in the gel were transferred to polyvinylidene difluoride (PVDF) membranes (Millipore, USA). After blocking in 5% bovine serum albumin (BSA) solution, the membranes were incubated with primary antibodies: CD163 (ab182422, abcam, USA), TfR (ab84036, abcam, USA), LRP1 (ab92544, abcam, USA), ADAM10 (14194, CST, USA), and caspase‐3 (9662, CST, USA) at 4 °C overnight. Then, the membranes were incubated with the corresponding secondary antibodies at room temperature (RT) for 1 h. Films were developed using Western chemiluminescent horseradish peroxidase (HRP) substrate (Millipore, USA) and evaluated with ChemiDoc imaging system (Bio‐Rad, USA).

### Membrane Derivation and Synthesis of Engineered ApoEVs

The engineered apoEVs were prepared according to a previous work.^[^ ^46^ ^]^ Briefly, the apoEVs were subjected to hypotonic treatment by resuspension in a hypotonic lysis buffer consisting of 10 mm tris (pH 7.4), 10 mm MgCl2, and 1 mm phenylmethylsulfonyl fluoride at 4 °C for 1 h, followed by gentle sonication for 5 s (VCX 130 PB, Sonics, USA). After centrifugation at 100 g for 10 min to remove the debris, the apoEV membranes were concentrated by centrifugation at 10 000 g for 10 min and then washed in water at least three times until the intracellular components were removed, which was verified by fluorescence microscopy. The resulting apoEV membranes were dispersed in water and used for subsequent experiments. Engineered apoEVs were synthesized using a sonication method. Briefly, nanoparticle cores (MSNs) were prepared by the classical CTAB‐templated, base‐catalyzed sol‐gel method according to previous work.^[^ ^44^ ^]^ For apoEV membrane coating, 1 mg of MSNs were mixed with 2 mg of apoEV membranes, and then the suspension was sonicated for 2 min intermittently and gently in a bath sonicator (SY25‐12, Shengyuan Supersonic, China).

### Engineered ApoEVs Characterization

Engineered apoEVs were measured for hydrodynamic size and surface zeta potential with DLS (ZEN 3600 Zetasizer, Malvern). The morphology of engineered apoEVs was determined by TEM (TECNAI Spirit, FEI). Surface markers on apoEV, MSN, and apoEV^@MSN^ were examined by western blotting as described above.

### Membrane Protein Retention

Western blotting analysis was performed to identify protein retention during the manipulation of engineered apoEVs. Twenty microliters of sample were loaded into each well of a 10% SDS‐polyacrylamide gel in a Bio‐Rad Electrophoresis System. Protein staining was accomplished using Coomassie Blue Fast Staining solution, and the gels were destained in deionized water at 4 °C overnight before imaging. For Western blot analysis, PVDF membranes were blocked with 5% BSA solution for 1 h and then incubated with antibodies against CD163, TfR, LRP1 and ADAM10. Films were developed using western chemiluminescent HRP substrate with ChemiDoc imaging system (Bio‐Rad, USA).

### Cellular Uptake of ApoEV@MSN

To verify the uptake of engineered apoEVs by macrophages in vitro, macrophages were treated with PKH26‐apoEV membrane (50 µg mL^−1^), RhB‐MSNs, and RhB‐apoEV@MSN for 3 h, and PBS was used as a control. Then, the cells were fixed with 4% PFA overnight and blocked at 37 °C for 30 min. After incubation with anti‐F4/80 antibody (ab6640, abcam, USA) and FITC–anti‐rat secondary antibody, the nuclei were counterstained with Hoechst 33342. Fluorescence imaging was performed by CLSM. Macrophages were also treated with RhB‐apoEV@MSN (25 µg mL^−1^) at different time points.

### MiRNA Sequencing Experiment

Total RNA was extracted using mirVana miRNA Isolation Kit (Ambion, USA) according to the manufacturer's instruction. Quantitation was carried out using the Nanodrop 2000 (Thermo Fisher Scientific Inc., USA). RNA integrity was assessed by Agilent 2100 Bioanalyzer (Agilent Technology, USA). One micrograms of total RNA of each sample was used for the small RNA library construction using NEBNext Small RNA Library Prep Set for Illumina kit (NEB#E7330S, NEB, USA) according to the manufacturer's recommendations. Briefly, total RNA was ligated to adapters at each end. Then the adapter‐ligated RNA was reverse transcribed to cDNA and performed PCR amplification. The PCR products ranging from 140–160 bp were isolated and purified as small RNA libraries. The libraries were finally sequenced using the Illumina Novaseq 6000 platform. The miRNA sequencing and analysis were conducted by OE Biotech Co., Ltd. (Shanghai, China). Differentially expressed miRNAs were calculated and filtered with the threshold of *q* value < 0.05 and FC > 2 or FC < 0.5. While the q value was calculated with DEG algorithm in the R package for experiment with biological replicates.

### MiRNA Loading and GSH‐Triggered Release from MSNs^miR‐146a^

The loading of miR‐146a on the MSNs was achieved via electrostatic interactions as previous description.^[^ ^47^ ^]^ Briefly, MSNs (50 mg) and (3‐mercaptopropyl)‐trimethoxysilane (200 µL) were completely dispersed in 20 mL of ethanol by sonication for 10 min (100 W). The solution was stirred overnight under nitrogen at 60 °C to obtain MSNs‐SH. Next, 225 mg of 2,2′‐dithiodipyridine and 145 mg of 2‐dimethylaminoethanethiol hydrochloride were added to the above solution to continue the reaction for 12 h. The mixture was further centrifuged, washed several times with ethanol, and dried under vacuum for 20 h to obtain the DMA‐grafted MSNs with disulfide bonds (MSNs+). Furthermore, 0.06 mg of MSNs+ and 15 nmol of miR‐146a were mixed in 60 µl of nuclease‐free water at 4 °C by repeated aspiration for 1 h. The mixture was further centrifuged and washed three times with nuclease‐free water to obtain miR‐146aMSNs. MSNs^Cy5‐miR‐146a^ were produced by the same method. The encapsulation capacity of MSNs for miR‐146a was calculated as follows: Encapsulation efficiency = Amount of ^miR‐146a^ in MSNs/Initial amount of ^miR‐146a^

The major elements (C, N, O, S, and Si) in the MSNs+ were identified through XPS. The characteristic peaks of the elements S and N are magnified to demonstrate the successful modification of MSN+ (ESCALAB Xi+, Thermo Fisher Scientific). The DLS measurements and infrared spectroscopy were performed by a Malvern Zetasizer Nano Series and Avatar 320 FT‐IR spectrometer, respectively, to further investigate the stepwise modification.

### Fabrication of RBCs Driven Engineered ApoEVs

Construction of the apoEV^miR‐146a@MSN^‐RBC was realized by the attachment of apoEV^miR‐146a@MSN^ to RBCs through the PN‐TER119 binding complex. For PN‐TER119 synthesis, PN was conjugated to the amino end of the TER‐119 by NHS‐amino coupling chemistry.^[^ ^23^ ^]^ Briefly, 100 µL of 0.5 mg mL^−1^ TER119 (Proteintech) in 100 mm carbonate buffer (pH 8) were mixed with 20 µL (3 equivalents) of PN (50 µm in DMSO) and allowed to react for 3 h at room temperature. Excess unreacted PN was removed by dialysis (3000 Da). PN‐TER119 dissolved in deionized water was analyzed by matrix‐assisted laser desorption ionization time‐of‐flight mass spectrometry (MALDI‐TOF MS) (Bruker ultraflextreme MALDI‐TOF/TOF, USA). To combine apoEV^miR‐146a@MSN^ with PN‐TER119, 100 µL apoEV^miR‐146a@MSN^ (53 µg mL^−1^) and 100 µL PN‐TER119 (200 µg mL^−1^) were gently mixed for 30 min. The mixture was then ultracentrifuged at 16 000 g for 30 min to collect PN‐TER119 modified apoEV^miR‐146a@MSN^.

Isolation of RBCs was performed via the methods previously described.^[^ ^48^ ^]^ Briefly, whole blood from C57BL/6J mice was collected in anti‐coagulant tubes. Blood was then spun at 2000 g for 20 min at 4 °C. The supernatant was discarded. Isolated RBCs were washed extensively with 1 × Dulbecco's Phosphate Buffered Saline (DPBS), centrifuged (500 g, 15 min, 4 °C). For attachment of apoEV^miR‐146a@MSN^ to RBCs, RBCs were incubated with PN‐TER119 modified apoEV^miR‐146a@MSN^ at ratios between 1:20 and 1:30 for 1 h under constant rotation at 37 °C in PBS.^[^ ^49^ ^]^ ApoEV^miR‐146a@MSN^/RBC solution was filtered with a 1 µm filter pore size membrane to remove unattached apoEV^miR‐146a@MSN^.

### Characterization of RBCs Driven Engineered ApoEVs

The morphology of apoEV^miR‐146a@MSNs^‐RBC was determined by SEM (Hitachi, Japan), TEM (JEOL, Japan) or CLSM (Nikon, Japan). Samples for scanning electron microscopy were fixed in 2.5% glutaraldehyde and 2.0% paraformaldehyde in 1 m cacodylate buffer, pH 7.4, overnight at 4 °C. RBCs were allowed to adsorb onto poly‐l‐lysine‐treated coverslips for 1 h, then washed several times in the same buffer. Samples were post‐fixed in 2.0% osmium tetroxide for 1 h, washed again in buffer and dehydrated in a graded ethanol series. Samples were treated with several changes of hexamethyldisilazane (HMDS) and allowed to air dry prior to mounting and sputter coating with gold/palladium. SEM images were obtained in S4800 scanning electron microscope. In the TEM observation, a total of 4 mL of the apoEV^miR‐146a@MSN^ solution at a concentration of 1 mg mL^−1^ was deposited onto a carbon‐coated 400‐square mesh copper grid. Ten minutes after the sample was deposited, the grid was rinsed with ten drops of water. A drop of 1% phosphotungstic acid was added to the grid to conduct the negative staining. The grid was subsequently dried naturally and visualized using the TEM. In the CLSM observation, 5‐(Octadecanoylamino) fluorescein (ex/em: 490/520 nm, abcam, USA) and the DiD (ex/em: 646/663 nm; Thermo Fisher Scientific, USA) were used to fluorescently visualize RBCs and engineered apoEVs, respectively. Finally, the immunofluorescence images were obtained by CLSM.

Percentage of the RBCs labeled positive for apoEV^miR‐146a@MSN^ before and after injection were validated using flow cytometry analyses. Briefly, 5‐(Octadecanoylamino) labeled RBCs and DiD labeled apoEV^miR‐146a@MSN^ were analyzed using a CytoFLEX Flow Cytometry System (Beckman Coulter, California). For each experiment, events were recorded using a 520 nm emission filter for 5‐(Octadecanoylamino) and 663 nm emission filter for DiD.

### Deformability Analysis of ApoEV^miR‐146a@MSN^‐RBC

Passive deformability and stability of the apoEV^miR‐146a@MSN^‐RBC were investigated by flowing apoEV^miR‐146a@MSN^‐RBC through microchannels with a width of 4 µm. The polydimethylsiloxane (PDMS)‐based microfluidic chips were made by soft lithography replica molding method.^[^ ^50^ ^]^ The apoEV^miR‐146a@MSNs^ were labeled with DiD and RBCs were labeled with 5‐(Octadecanoylamino) fluorescein. The fluorescently labeled apoEV^miR‐146a@MSNs^‐RBC were manually injected through the microchannels using a syringe. The motion of apoEV^miR‐146a@MSN^‐RBC was monitored by CLSM.

### In Vitro Blood Flow Model

A microfluidic device (height 0.3 mm, width 1.5 mm, length 46 mm) was purchased from BEOnChip (Zaragoza, Spain). The inlet and outlet of the microfluidic device are connected to the tubes, which are plugged into the pump through Teflon tubing. Fluid was driven by Flow‐EZ (Fluigent, Germany). Flow‐EZ is a pressure controller that is used to accurately control the air pressure of a medium reservoir to drive fluids through the microfluidic device.

### In Vivo Circulation Time Assay

To evaluate the in vivo circulation time, 150 µL of RhB‐apoEV^miR‐146a@MSN^ or RhB‐apoEV^miR‐146a@MSN^‐RBC were injected into the normal mice intravenously. Blood was collected at 1, 6, and 24 h following the injection, and the fluorescence intensity of RhB was measured using a multimode plate reader (HH3400, PerkinElmer; excitation = 540 nm/emission = 625 nm).

### In Vivo Fluorescence Tracing of ApoEVs or Engineered ApoEVs

For fluorescence tracing of apoEVs or engineered apoEVs, DiD‐apoEVs or DiD‐engineered apoEVs were administrated into mice via intravenous injection. After certain time points (1, 6 and 24 h), mice were euthanized, and the organs were excised for analysis. Fluorescent images were obtained with IVIS Spectrum Imaging System (PerkinElmer, USA). The luminescent signal was evaluated manually using Living Image Software (PerkinElmer, USA).

### Histopathological Evaluation

The liver and spleen samples were routinely processed for histologic analysis, and sections (5 µm thick) were stained with hematoxylin and eosin (H&E). After staining, the sections were evaluated with Pannoramic MIDI automated digital slide scanner (3DHistech, Budapest). Images were obtained with CaseViewer 2.3 analysis system (Servicebio, China). The tissue injury degree was determined by the histological scoring analysis according to previous work.^[^ ^12^ ^]^ The assessment of liver injury was expressed as the sum of the individual score grades of 0 (normal), 1 (slight injury), 2 (moderate injury), 3(strong injury) and 4 (maximum injury) for each of the following five categories: inflammation infiltration, cytoplasm vacuolization, nuclear condensation, hemorrhage, and hepatocyte necrosis. To evaluate the splenic histological changes, a semi‐quantitative scoring system was used. The histopathological changes in spleen were classified based on the severity of three histological criteria: architecture loss, necrotic cells, and inflammation. The histopathological changes were graded on a scale as follows: 0 (organized with periarteriolar sheath, germinal centers, and distinct mantle and marginal zone), 1 (slightly disorganized, with some hyperplastic or hypoplastic change leading to the loss of definition of some regions of the white pulp), 2 (moderately disorganized, with evident white pulp, but the regions are barely individualized or indistinct), 3 (intensely disorganized, with a follicular structure little distinguishable from the red pulp and the T cell area).

### Biocompatibility Test

MTT assay was adopted to examine the cytotoxicity of engineered apoEVs in vitro. Briefly, macrophages or A549 cells were seeded in 96‐well plates at a concentration of 10^3^ cells per well. After cell adhesion, MSNs (50 µg mL^−1^) and various concentrations of engieered apoEVs (10, 50, 100, and 150 µg mL^−1^) were added to the plate. After 24 h of treatment, cell viability was detected by the standard MTT assay according to the instructions. For safety evaluation of engineered apoEVs in vivo, ApoEV^miR‐146a@MSN^ (50 µg of membrane protein) or ApoEVmiR‐146a@MSN–RBC (50 µg of membrane protein) was injected into normal mice every other day for a total of five times (n = 6). At the endpoint, the blood was obtained to carry out the hematology and serum biochemistry tests. The major organs were harvested to conduct H&E staining. Samples from PBS‐treated mice were used as controls.

### Statistical Analysis

Data are presented as means ± s.d, and analyzed using the SPSS 19.0 (IBM, USA). Statistical analysis was performed by unpaired two‐tailed Student's *t*‐test, one‐way analysis of variance (ANOVA) with Tukey's post‐hoc test or Kruskal–Wallis test. Survival functions were compared using the log‐rank test. The difference between groups was considered statistically significant for ^*^ *p* < 0.05, ^**^ *p* < 0.01. Graph analysis was performed using GraphPad Prism 7.00 (GraphPad Software, USA).

## Conflict of Interest

The authors declare no conflict of interest.

## Author Contributions

Y.L., G.Q., and G.D. contributed equally to this work. Y.L. and S.L. designed, performed, and interpreted the experiments and wrote the manuscript. Q.L. performed bacterial experiments, animal experiments, histopathological studies and collected data. G.D. and H.K. contributed to the data acquisition and interpretation. L.B., F.D., and G.X. contributed to data analysis. L.R. design and created the RBC attachment. S.L. and Y.L developed the original concept. S.L., C.Y., and Z.Z conceived the study and supervised the experiments. S.L, Y.L., and M.D. were responsible for the manuscript organization.

## Supporting information

Supporting Information

## Data Availability

The data that support the findings of this study are available from the corresponding author upon reasonable request.
