# Supplementary material for: Engineered Extracellular Vesicles Driven by Erythrocytes Ameliorate Bacterial Sepsis by Iron Recycling, Toxin Clearing and Inflammation Regulation
Source: Adv Sci (Weinh). 2024 Jan 21;11(13):2306884. doi: 10.1002/advs.202306884 (PMC10987154; doi:10.1002/advs.202306884)
Supplement: Supplementary file 1 — Supporting Information [file ADVS-11-2306884-s001.pdf]

ADVANCED SCIENCE
Open Access

Supporting Information

for *Adv. Sci.*, DOI 10.1002/advs.202306884

Engineered Extracellular Vesicles Driven by Erythrocytes Ameliorate Bacterial Sepsis by Iron Recycling, Toxin Clearing and Inflammation Regulation

Yan Li, Guanlin Qu, Geng Dou, Lili Ren, Ming Dang, Huijuan Kuang, Lili Bao, Feng Ding, Guangzhou Xu, Zhiyuan Zhang, Chi Yang* and Shiyu Liu**

Supplementary Figures

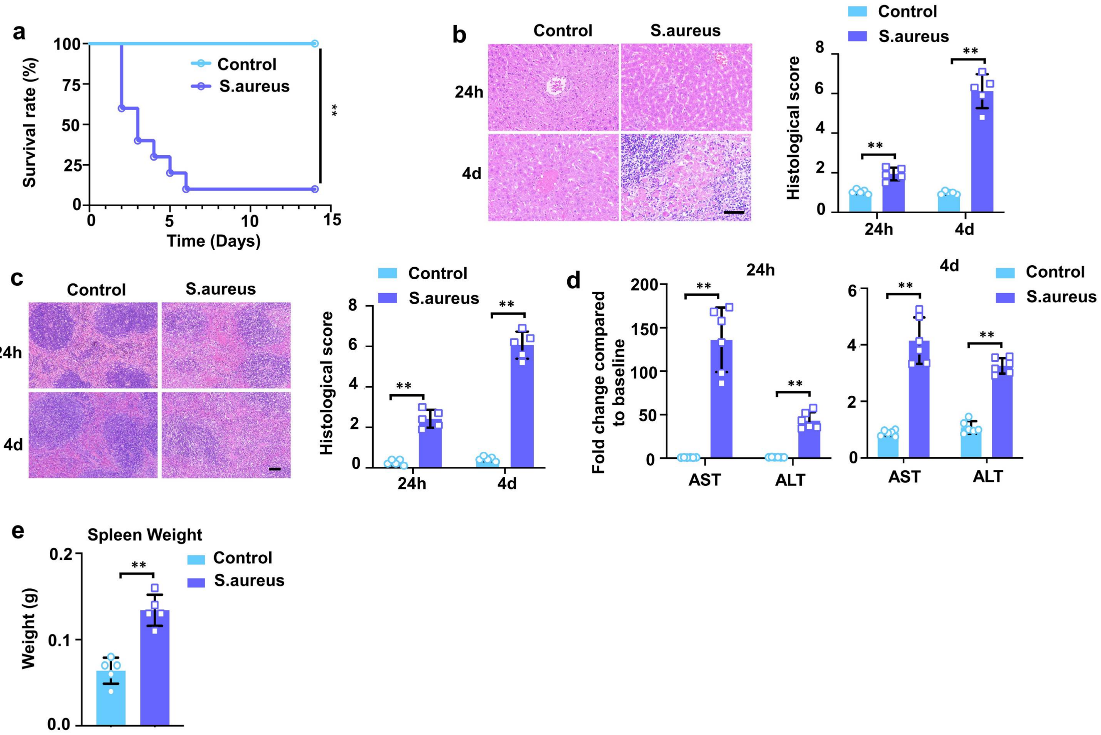

Figure S1. Characterization of S.aureus-infected mice. a, Survival rate of uninfected mice and infected mice. **b**, H&E staining of representative liver sections and the associated histological scores at 24 hours or day 4 after infection. Scale bar, 50 μm. n = 5 mice. **c,** H&E staining of representative spleen sections and the associated histological scores at 24 hours or day 4 after infection. Scale bar, 50 μm. n = 5 mice. **d**, ALT and AST levels in the serum of uninfected mice and infected mice. n = 5 mice. **e**, The spleen weight at day 4 after infection. n = 5 mice. All the experiments were repeated at least twice. For **a**, statistical significance was assessed by the log-rank test. For **b, c, d** and **e,** data are represented as the mean ± s.d., statistical significance was assessed by unpaired two-tailed Student's t test. **p < 0.01

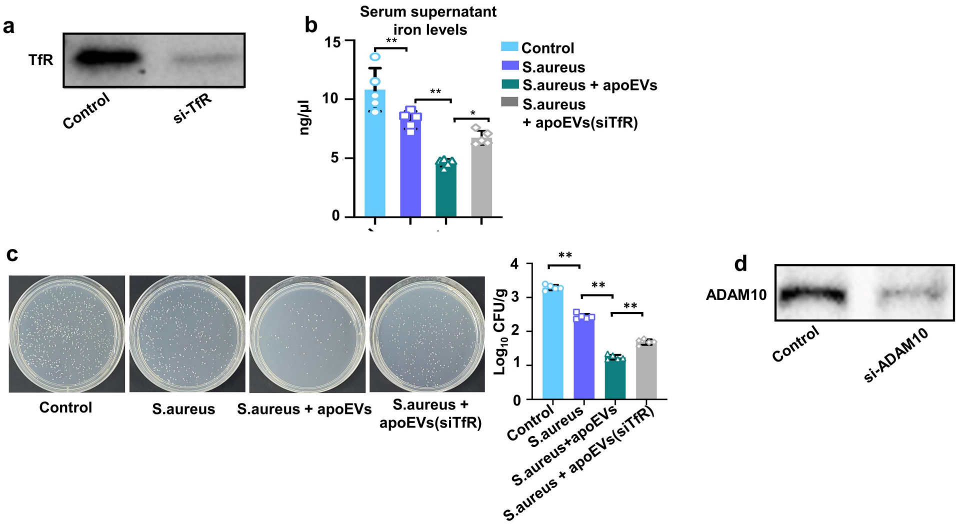

Figure S2. Inhibition of the TfR or ADAM10 expression in macrophage-derived apoEVs via siRNA. a, Western blot analysis of TfR in apoEVs isolated from macrophage pretreated with TfR siRNA. **b**, Total iron levels in the serum supernatant. n = 5. **c**, Viable count of S.aureus in serum supernatant. n = 5. **d**, Western blot analysis of ADAM10 in apoEVs isolated from macrophage pretreated with ADAM10 siRNA. All the experiments were repeated at least twice. Data are presented as mean±SD. Statistical significance was assessed by one-way ANOVA with Tukey's post-hoc test. *$p < 0.05$, **$p < 0.01$.

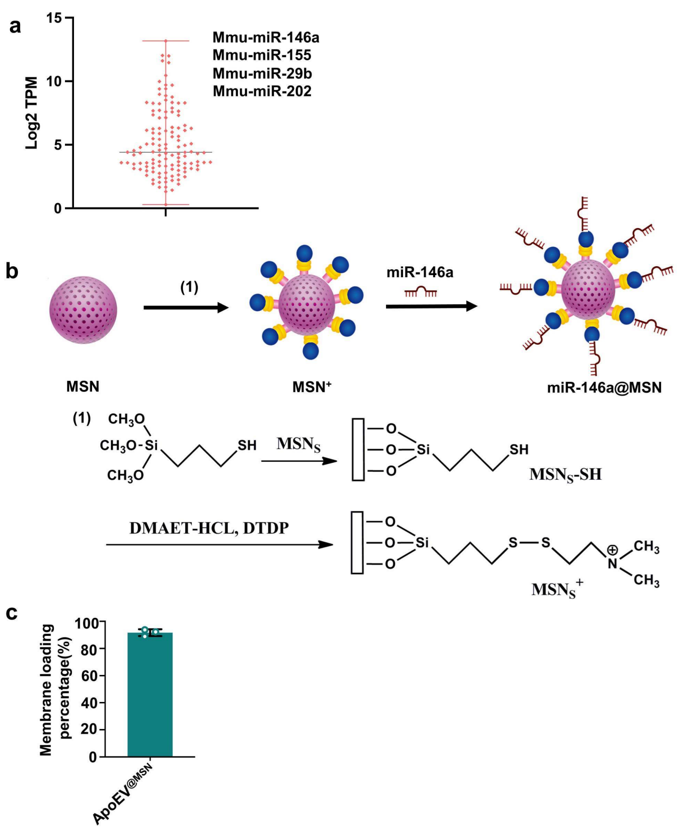

Figure S3. Characterization of engineered apoEVs. a, Graph depicting the top 4 upregulated miRNAs detected in apoEVs from S. aureus infected mice. **b,** Design of stimuli-responsive engineered apoEVs for RNA loading and intracellular release. The loading of miR-146a was performed by electrostatic assembly between negatively charged miR-146a and positively charged MSN with GSH-responsive S-S bonds. **c**, The membrane loading percentage of ApoEV$^{@MSN}$ (n=3).

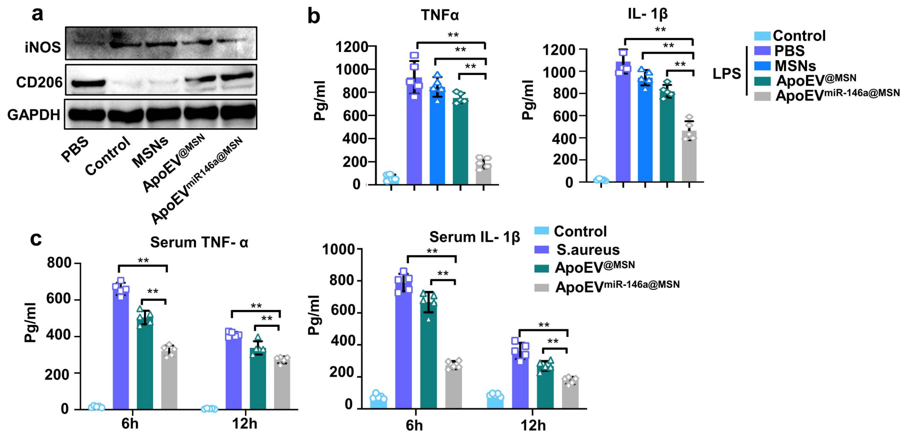

Figure S4. The immunoregulatory effect of engineered apoEVs loaded with miR-146a. **a**, Western blot analysis of the phenotype markers in LPS-stimulated macrophages after different treatments. **b,** Detection of the inflammatory factors (TNF-α and IL-1β) released by macrophages by ELISA. n = 5. **c**, Concentration profiles of TNF-α and IL-1β in the serum of S. aureus infected mice treated with different groups. n = 5 mice. All results are representative of the data generated in at least two independent experiments and are presented as mean ± SD. Statistical significance was assessed by one-way ANOVA with Tukey's post-hoc test. *p < 0.05, **p < 0.01

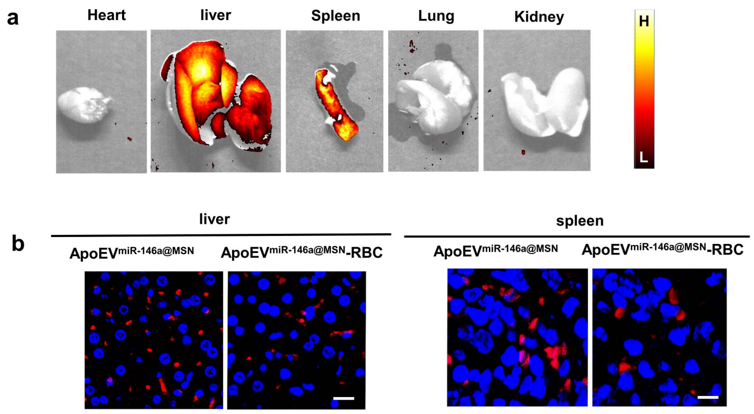

Figure S5. The biodistribution of engineered apoEV$^{miR\text{-}146a@MSN}$. a, *Ex vivo* fluorescent images of various organs in mice injected with DiD-labeled apoEV$^{miR\text{-}146a@MSN}$. n=3 mice. **b**, The uptake of unmodified or apoEV$^{miR\text{-}146a@MSN}$-RBC (red) in liver or in spleen stained with Hoechst (nucleus; blue). Scale bar, 20μm.

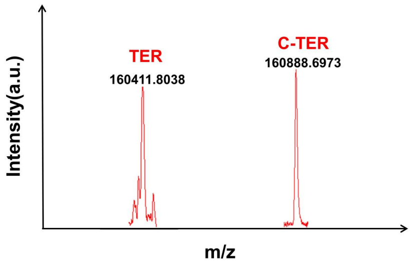

Figure S6. MALDI-TOF mass spectra of TER and C-TER. After modification, the molecular weight increased by approximate 477 Daltons, which corresponded to 2 N-succinimidyl palmitate molecules per TER.

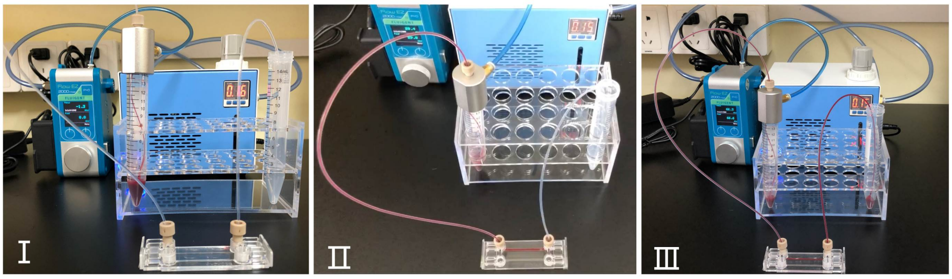

Figure S7. Schematics of the flow experiment. Inlet and outlet of flow channel are connected to the tube via teflon tubing. The flow channel is 43 mm long, 375 height µm and 1.5mm wide. The flow control was accomplished using the Flow EZ™ system of FLUIGENT.

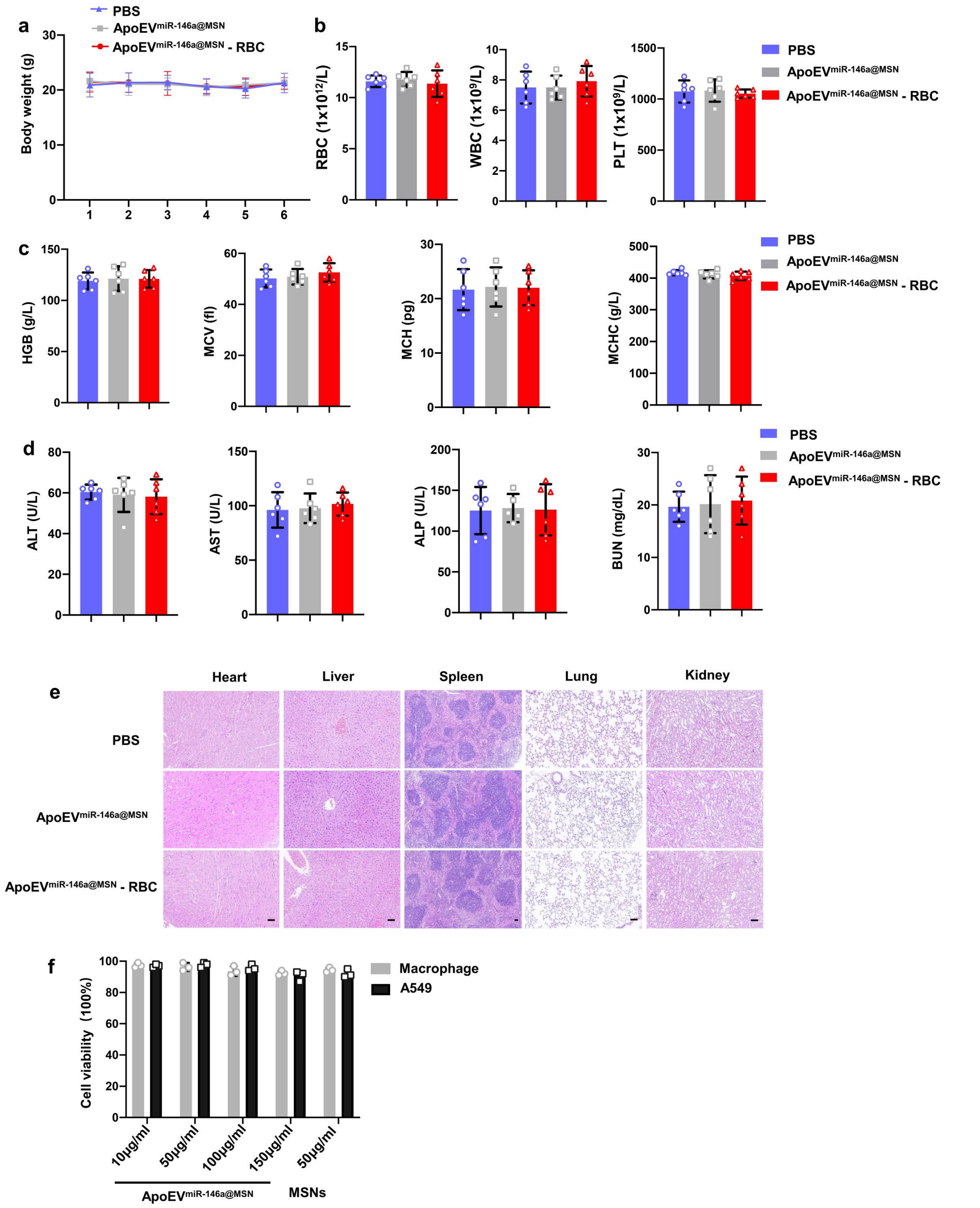

Figure S8. Biocompatibility and safety of engineered apoEVs. a, The body weight of mice in each group (n=6). **b**, Complete cell counts in the PBS and treated groups (n=6). RBC, red blood cells; WBC, white blood cells; PLT, platelets. **c**, Blood routine examination parameters after ApoEV$^{miR-146a@MSN}$/ApoEVmiR-146a@MSN – RBC treatment (n=6). HGB, hemoglobin; MCV, mean corpuscular volume; MCH, mean corpuscular hemoglobin; MCHC, mean corpuscular hemoglobin concentration. **d**, Serum biochemical indicators after ApoEV$^{miR-146a@MSN}$/ApoEVmiR-146a@MSN–RBC treatment (n=6). ALT, alanine aminotransferase; AST,

aspartateaminotransferase; ALP, alkaline phosphatase; BUN, blood urea nitrogen. **e**, H&E staining of major organs in different groups; scale bar, 100 μm. **f**, Cytotoxicity of engineered apoEVs in vitro. Viability of macrophages and A549 cells incubated with various concentrations of engineered apoEVs and MSNs detected by MTT assay (n=3). All results are representative of the data generated in at least three independent experiments and are presented as mean ± SD.

miR-146a/MSN (μmol/mg)	Encapulation efficiency (%) (miR-146)
0.1/1	99.52
0.2/1	99.67
0.3/1	99.83

Table S1. Encapsulation capacity of functional molecules on MSN$^{miR\text{-}146a}$. Encapsulation efficiency= (encapsulated quality of cargos)/(initial quality of cargos).

Abbreviation	Construction	Loading cargos
MSN	Mesoporous silica nanoparticle	None
$MSN^{miR-146a}$		miR-146a
$ApoEV^{@MSN}$	Engineered apoEVs	None
$ApoEV^{miR-146a@MSN}$		miR-146a

Table S2. Abbreviations in this paper.
